# An Alternative and Effective HIV Vaccination Approach Based on Inhibition of Antigen Presentation Attenuators in Dendritic Cells

**DOI:** 10.1371/journal.pmed.0030011

**Published:** 2006-01-03

**Authors:** Xiao-Tong Song, Kevin Evel-Kabler, Lisa Rollins, Melissa Aldrich, Feng Gao, Xue F Huang, Si-Yi Chen

**Affiliations:** 1Center for Cell and Gene Therapy, Baylor College of Medicine, Houston, Texas, United States of America; **2**Department of Molecular and Human Genetics, Baylor College of Medicine, Houston, Texas, United States of America; **3**Department of Immunology, Baylor College of Medicine, Houston, Texas, United States of America; **4**Duke Human Vaccine Institute, Duke University Medical Center, Durham, North Carolina, United States of America; **5** Department of Pediatrics, Baylor College of Medicine, Houston, Texas, United States of America; National Institutes of HealthUnited States of America

## Abstract

**Background:**

Current efforts to develop HIV vaccines that seek to stimulate immune responses have been disappointing, underscoring the inability of natural immune responses to control HIV-1 infection. Here we tested an alternative strategy to induce anti-HIV immune responses by inhibiting a host's natural immune inhibitor.

**Methods and Findings:**

We used small interfering RNA (siRNA) to inhibit suppressor of cytokine signaling (SOCS) 1, a key negative regulator of the JAK/STAT pathway, and investigated the effect of this silencing on the ability of dendritic cells (DCs) to induce anti-HIV-1 immunity. We found that SOCS1-silenced DCs broadly induced enhanced HIV-1 envelope (Env)-specific CD8^+^ cytotoxic T lymphocytes and CD4^+^ T helper cells, as well as antibody responses, in mice. Importantly, SOCS1-silenced DCs were more resistant to HIV Env-mediated suppression and were capable of inducing memory HIV Env-specific antibody and T cell responses. SOCS1-restricted signaling, as well as production of proinflammatory cytokines such as interleukin-12 by DCs, play a critical role in regulating the anti-HIV immune response. Furthermore, the potency of HIV DNA vaccination is significantly enhanced by coimmunization with SOCS1 siRNA expressor DNA.

**Conclusions:**

This study demonstrates that SOCS1 functions as an antigen presentation attenuator to control both HIV-1-specific humoral and cellular responses. This study represents the first, to our knowledge, attempt to elicit HIV-specific T cell and antibody responses by inhibiting a host's antigen presentation attenuator, which may open a new and alternative avenue to develop effective therapeutic and prophylactic HIV vaccines.

## Abstract 2

Si-Yi Chen and colleagues describe a possible alternative strategy for HIV vaccination in mice involving suppressor of cytokine signaling (SOCS) in dendritic cells.

## Introduction

Despite extensive efforts, no effective human immunodeficiency virus (HIV) vaccine has emerged or is on the horizon [1,2]. Increasing evidence indicates that the host's natural immunity has a major, albeit usually insufficient, role in limiting HIV-1 infection. CD8^+^ cytotoxic T cells (CTLs) are the main mediators of viral control, as demonstrated by the dramatic increase in viremia in animal models after depletion of CD8^+^ T cells [3,4]. Although definitive evidence for a protective role of antibodies is lacking, a number of monoclonal antibodies generated from infected individuals have broadly neutralizing activities against primary HIV-1 isolates [5]. Antibodies to the HIV-envelope (Env) protein, gp120, protect animals such as monkeys and SCID-peripheral blood lymphocyte mice from HIV or SIV infection [6,7]. Thus, there is a growing consensus that an effective HIV immunization approach should be capable of inducing vigorous protective CTL as well as antibody responses [5,8–11].

Dendritic cells (DCs), the most potent antigen-presenting cells (APCs), mediate innate and adaptive immunity against viral infection by providing proinflammatory cytokines and by processing and presenting antigens to T cells [12]. DCs use Toll-like receptors (TLRs) to recognize conserved microbial structures such as lipopolysaccharide (LPS). TLR signaling promotes DC maturation by activating mitogen-activated protein kinase and nuclear factor-κB (NF-κB), which then mediate the expression of various cytokines, resulting in the induction of innate and adaptive immunity [13,14]. Hence, exploiting the full immunostimulatory potential of DCs is likely the key to achieving an effective immune response to prevent or control HIV infection.

Suppressor of cytokine signaling (SOCS) 1 is a key negative regulator of signaling of cytokines, such as interferon (IFN)-γ, interleukin (IL)-2, IL-6, IL-7, IL-12, and IL-15, through the inhibition of the Janus kinases (JAKs) in many lineages of immune cells [15,16]. Although the SOCS family includes eight members, each member plays a unique role in attenuating cellular signaling [15,16]. SOCS1 binds to the JAK activation loop as a pseudosubstrate inhibitor through its Src-homology 2 domain and targets JAK2 for degradation*,* leading to the inhibition of the molecule, signal transducer and activator of transcription (STAT) [15,16]. SOCS1-deficient (SOCS1^−/−^) mice die neonatally, with severe systemic inflammation and aberrant T cell activation, mainly as a result of unbridled cytokine signaling [17–19]. Such SOCS1^−/−^ mice are also hypersensitive to LPS, and SOCS1^−/−^ DCs show a more mature phenotype than do wild-type (WT) DCs and induce autoreactive antibody production [20,21], suggesting that SOCS1 plays a role in regulating DC functions by inhibiting the JAK/STAT pathway and the TLR signaling pathway directly or indirectly [15,22,23]. We have previously shown that SOCS1 plays a critical role in regulating the antigen presentation by DCs, and DCs in which SOCS1 expression is silenced by small interfering RNA (siRNA) induce enhanced CTL responses against tumor-associated antigens [24]. In agreement with this result, Hanada et al. recently reported that immunization with SOCS1^−/−^ DCs derived from SOCS1 genetic knockout mice induced a hyper CD4^+^ T helper cell (Th1)-type immune response and antitumor activities [25].

Many attempts to develop HIV vaccines have sought to stimulate immune responses by manipulating HIV antigens and delivery systems and by using various adjuvants [5,8–10]. These efforts have been disappointing and have underscored the inability of natural immune responses to control HIV-1 infection in most infected or immunized individuals. We therefore tested an alternative hypothesis that anti-HIV immune responses can be enhanced by silencing the host's natural immune inhibitors. In this study, we used siRNA to silence SOCS1 and investigated the effect of this silencing on the ability of DCs to induce anti-HIV-1 antibody and T cell responses in mice.

## Methods

### Cytokine and Antibody ELISAs

Cytokine levels in cell culture supernatant were quantified by ELISA analysis (BD Biosciences, San Diego, California, United States), according to the manufacturer's instructions. To determine gp120-specific antibody and subclass titers, we coated gp120 proteins (5 μg/ml in carbonate buffer [pH 9.6]) overnight at 4 °C, adding 12-fold serial dilutions of sera in PBS-5% FBS to the wells for 1 h at room temperature. After eight washes, biotinylated anti-mouse antibodies (anti-mouse IgM, IgG, IgG1, IgG2a, IgG2b, or IgG3) were added to the wells for 1 h at room temperature. Streptavidin-HRP was used as a peroxidase substrate. The reaction was stopped by addition of 50 μl of 2 M H_2_SO_4_. Optical densities were read at 450 nm on a BioAssay Reader (PerkinElmer, Wellesley, California, United States). The results are expressed as reciprocal endpoint titers, determined from a scatter plot with OD values on the y-axis and dilution-1 on the x-axis, for which the x-axis scale was logarithmic. After the data were plotted, a logarithmic curve fit was applied to each individual dilution series, and the point where the curve fit intersects the positive-negative cutoff value was determined. The cutoff value was calculated for each antibody isotype as the mean (± 3 standard deviations) of all dilutions from control mouse sera. All samples tested in each experiment were assayed at the same time.

### Transduction of Bone Marrow-Derived DCs with Lentiviral or Adenoviral Vectors

Recombinant lentiviral vectors, LV-SOCS1-siRNA and LV-GFP-siRNA, were produced and titrated, as described previously [24]. A recombinant adenoviral vector (Ad-IL-12) expressing a biological active mouse IL-12 (a fusion protein of p35 and p40) was purchased from InvivoGen (San Diego, California, United States) and produced according to the manufacturer's instruction. Adenoviruses were titrated using Adeno-X Rapid Titer Kits (BD Bioscience). Mouse bone marrow (BM)-derived DCs were generated by culturing with granulocyte-macrophage colony-stimulating factor (GM-CSF) and IL-4, and transduced by lentiviral or adenoviral vectors as described previously [24].

### T Cell Enzyme-Linked Immunospot Assays

Enzyme-linked immunospot (ELISPOT) assays of isolated CD4^+^ or CD8^+^ T cells were performed as described in our previous reports [24]. Recombinant gp120 protein-pulsed BM-DCs were used for T cell stimulation. An irrelevant protein (ovalbumin [OVA]; Sigma, St. Louis, Missouri, United States) was also used as a negative control. CD4^+^ and CD8^+^ T cells were isolated from splenocytes with MACS CD4 (L3T4) or MACS CD8 (Ly-2) MicroBeads (Miltenyi Biotec, Auburn, California, United States).

### B Cell Isolation and gp120 Antibody-Producing B Cell ELISPOT Assay

Single-cell suspensions prepared from spleens in complete RPMI 1640 medium were plated on plastic dishes for 1 h at 37 °C in 5% CO_2_ to remove adherent macrophages. Nonadherent cells were treated with anti-Thy1.2 and rabbit complement for 45 min at 37 °C to lyse T cells. The purity of the remaining B cells usually exceeded 90%. The B cell ELISPOT assay was performed by a modified method described before [26]. Briefly, 96-well nitrocellulose-base plates (Millipore Multiscreen PI, Billerica, Massachusetts, United States) were coated overnight with gp120 in PBS. The plates were washed six times with PBS and blocked with RPMI 1640 containing 10% FBS at 37 °C for 2 h. The isolated B cells were seeded into wells (5 × 10^5^ cells/well) and incubated for 20 h at 37 °C in 5% CO_2_. The cells were then removed by six washes with PBS containing 0.5% Tween 20 (Sigma). Biotinylated anti-mouse IgG (BD Pharmingen, San Diego, California, United States), diluted in PBS containing 0.5% FBS to 1 μg/ml, was added, and the mixture incubated at 37 °C for 2 h. The avidin:biotinylated enzyme complex ([ABC]; Vector Laboratories, Burlingame, California, United States) was added for an additional hour. Anti-gp120 IgG was detected after a 4-min reaction with AEC (3-amino-9-ethylcarbazole; Sigma). The results were evaluated by ZellNet Consulting (New York, New York, United States) with an automated ELISPOT reader system (Carl Zeiss, Thornwood, New York, United States), using KS ELISPOT 4.3 software.

### Quantitative Real-Time PCR Analysis of BAFF and APRIL

The relative expression of SOCS1 in transfected mouse BM-DCs was evaluated by quantitative real-time PCR. Total RNA was extracted from DCs, using Trizol reagent (Invitrogen, Carlsbad, California, United States), and 1.0 μg of total RNA for each sample was reverse transcribed with random hexamer primers and SuperScript First-Strand Synthesis kits (Invitrogen). Real-time 5′-nuclease fluorogenic PCR analysis was performed on an ABI 7900HT Sequence Detection System (Applied Biosystems, Foster City, California, United States) in 20-μl quadruplicate reactions with the equivalent of 5 ng of starting RNA material per reaction as template. The following primers were used for BAFF and APRIL: BAFF sense, 5′- TGCTATGGGTCATGTCATCCA-3′ and anti-sense, 5′- GGCAGTGTTTTGGGCATATTC-3′; APRIL sense, 5′- TCACAATGGGTCAGGTGGTATC-3′ and anti-sense, 5′- TGTAAATGAAAGACACCTGCACTGT-3′. TaqMan probe, forward and reverse primer for 18S were obtained from TaqMan Rodent 18S control reagents (Applied Biosystems). The PCR parameters were these recommended for the TaqMan Universal PCR Master Mix kit (Applied Biosystems), with BAFF, APRIL, and 18S reactions performed in separate tubes. BAFF and APRIL levels were normalized to 18S rRNA, while BAFF or APRIL expression (relative to the control value of mock-transfected, stimulated DCs) was calculated by the Comparative Ct method [24,27].

### CTL Assays

CD8^+^ CTL responses were assessed with a standard chromium release assay [24] that measures the ability of in vitro-restimulated splenocytes to lyse target cells. Splenocytes pooled from immunized mice were restimulated in vitro in RPMI-1640 containing gp120 proteins (20 μg/ml) for 4–6 d. Target cells pulsed with 20 μg/ml of gp120 protein overnight were labeled with ^51^Cr-sodium chromate solution for 90 min. Different numbers of effector cells were incubated with a constant number of target cells (1 × 10^4^/well) in 96-well V-bottom plates (200 μl/well) for 3 h at 37 °C. The supernatants (100 μl) from triplicate cultures were collected. Percent cell lysis was calculated as (experimental release – spontaneous release)/(maximum release – spontaneous release) × 100.

### T and B Cell Proliferation Assay

CD4^+^ or CD8^+^ T cells (1 × 10^6^ per well) and B cells (1 × 10^5^ per well) isolated as described above were cultured in complete medium in triplicate wells of 96-well plates with or without various stimuli. On the fourth day of culture, wells were pulsed with 1 μCi of [^3^H]-thymidine for 16 h. Plates were then harvested, and incorporated [^3^H]-thymidine was measured using a MicroBeta scintillation counter (TopCount NXT, Packard, Meriden, Connecticut, United States).

### DC Immunization

The recombinant soluble gp120 (SF162) protein, with a purity of over 95% and mostly in a monomeric form, was produced and purified from CHO cells and kindly provided by the National Institutes of Health AIDS Research and Reference Program and Chiron Corporation. On day 5 of BM culture, DCs derived from BM of WT mice or IL-12 receptor knockout (KO) mice were transduced with LV-SOCS1-siRNA or LV-GFP-siRNA at a multiplicity of infection (MOI) of 5 [24], and pulsed with proteins for 2 h. The transduced DCs were then stimulated with LPS (100 ng/ml, Sigma) or tumor necrosis factor α (TNFα) (50 ng/ml, Peprotech, Rocky Hill, New Jersey, United States) ex vivo for 24 h, washed with PBS, and injected into mice (Jackson Laboratory, Bar Harbor, Maine, United States) via a foot pad. IL-12 receptor KO mice (B6.129S1-*Il12rb1^tm1Jm^*) in a C57BL/6 background were purchased from the Jackson Laboratory. The immunized mice were treated with LPS, PolyI:C, or R837 (30 μg/mouse) or murine IL-12 (1 μg/mouse) intraperitoneally three times on days 1, 3, and 5 after each DC immunization.

### DNA Vaccination

The pSuper-SOCS1-siRNA expression vector was generated as described previously [24]. An HIV Env gp140 expression vector (pCMV/R-gp140CF) in which the gp120/gp41 cleavage site and fusion domain of HIV gp160 (codon usage-optimized HIV-1 strain JRFL) was deleted and the *gp140CF* gene was placed under control of the CMV promoter, was constructed. Endotoxin-free DNA was prepared with a DNA isolation kit from Qiagen (Valencia, California, United States), resuspended in endotoxin-free PBS (Sigma-Aldrich) at a final concentration of 1 μg/μl, and stored at −20 °C until used for injection. On the scheduled day of vaccination, 50 μg of *gp140CF* DNA or 200 μg of the mixture of *gp140CF* DNA (50 μg) and pSuper-SOCS1-siRNA expressor DNA (150 μg) [24] was injected into the quadriceps of each mouse [28,29]. The immunized mice were then treated with PolyI:C or R837 (30 μg/mouse) intraperitoneally three times on days 1, 3, and 5 after each DNA immunization [24].

### Statistical Analysis

We used the Student's t-test, and 95% confidence limits, to assess results for statistical significance, defined as *P* < 0.05. Results are typically presented as means ± standard error.

## Results

### Silencing of SOCS1 in DCs Enhances the HIV Env-Specific Antibody Response

**Figure 1:**
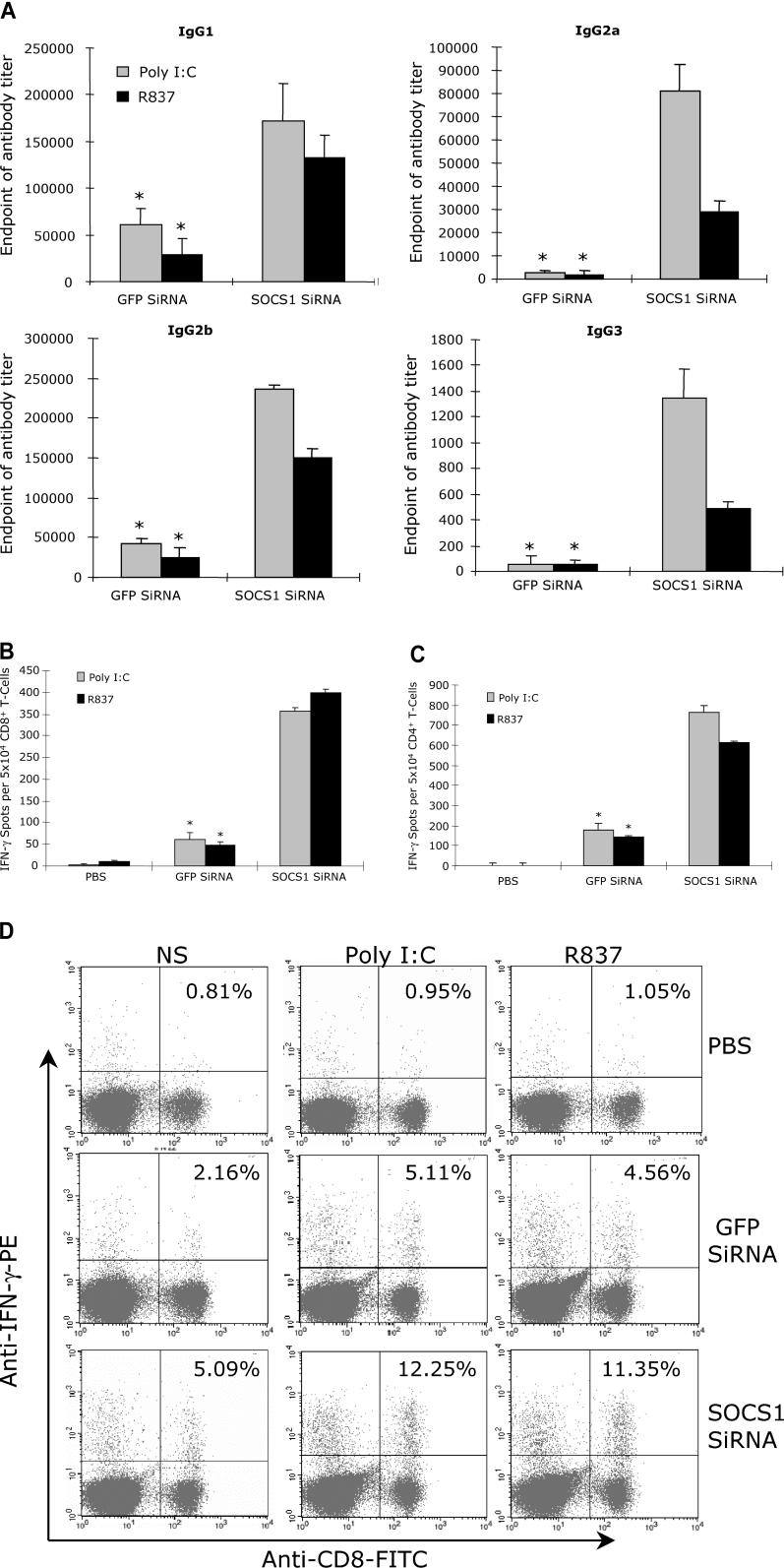
Enhanced gp120-Specific Antibody and T Cell Responses Induced by SOCS1-Silenced DCs Groups of C57BL/6 mice were immunized with gp120 (SF162) protein-pulsed, transduced BM-derived DCs (1 × 10^6^ cells/mouse) twice at a weekly interval, followed by PolyI:C or R837 stimulation (30 μg/mouse) in vivo three times on days 1, 3, and 5 after each DC immunization, and sera and splenocytes were collected from each group of mice 14 d later. HIV gp120-specific IgG subclass titers (A) from the pooled sera of each group (4–6 mice/group) were quantified by capture ELISA. CD8^+^ T cells (B) and CD4^+^ T cells (C) isolated from pooled splenocytes were used for IFN-γ ELISPOT assays stimulated with gp120 proteins. Intracellular IFN-γ staining of CD8^+^ T cells from the pooled splenocytes were also performed (D). Representative data from one of three experiments are presented. NS, no stimulation. *P < 0.01, LV-SOCS1-siRNA-DCs versus LV-GFP-siRNA-DCs.

We first investigated the effect of SOCS1 silencing on the ability of DCs to induce anti-HIV antibody responses. We used HIV Env for this study, since it can induce both cellular and neutralizing antibody responses. A recombinant lentiviral vector (LV-SOCS1-siRNA) that expresses SOCS1 siRNA and has the ability to down-regulate about 90% of SOCS1 mRNA in transfected cells and a control vector (LV-GFP-siRNA) were generated, as described previously [24]. Mouse BM-derived DCs were transduced with LV-SOCS1-siRNA or LV-GFP-siRNA, loaded with recombinant HIV gp120 proteins, and matured with TNFα ex vivo. Groups of mice were then immunized with the transduced DCs twice at a weekly interval. LV-SOCS1-siRNA-DCs elicited greater gp120-specific IgG responses than did the control LV-GFP-siRNA-DCs (Figure S1A and S1B). We further tested whether in vivo stimulation with a TLR agonist, PolyI:C or R837, could further enhance anti-gp120 immune responses, since SOCS1 is an inducible feedback inhibitor [15,16] and immune responses against tumor-associated antigens induced by SOCS1-silenced DCs were preferentially enhanced by in vivo stimulation with LPS in our previous study [24]. Groups of mice were then immunized with the transduced DCs twice at a weekly interval, followed by stimulation with a low dose of PolyI:C or R837 in vivo after each DC immunization. Figure 1 shows increases in HIV Env-specific antibody titers in all IgG subclasses in mice immunized with LV-SOCS1-siRNA-DCs, compared with the corresponding IgG subclasses in LV-GFP-siRNA-DC mice. In vivo stimulation with PolyI:C or R837 preferentially enhanced HIV Env-specific antibodies, especially IgG2 and IgG3, in mice immunized with LV-SOCS1-siRNA-DCs (Figure 1). The Env-specific antibody subclass profile showed a Th1-polarized IgG response, higher IgG2a (a subclass associated with a Th1 response [30]), induced by LV-SOCS1-siRNA-DCs. Similar results were obtained in repeated experiments. In addition, in vivo stimulation with LPS also significantly enhanced HIV Env-specific antibody titers in LV-SOCS1-siRNA-DC mice (Figure S2). We did not perform neutralizing assays, since mice are not an appropriate species for reliable testing of HIV neutralizing activities [5]. We further found that SOCS1 silencing enhanced antibody responses to other strains of HIV Env proteins and antigens such as OVA (unpublished data). These results demonstrate that HIV Env-specific antibody responses are enhanced by the silencing of SOCS1 in DCs, implying a critical role for SOCS1 in DCs in controlling antigen-specific antibody responses.

### Silencing of SOCS1 in DCs Enhances HIV gp120-Specific T Cell Responses

**Figure 2:**
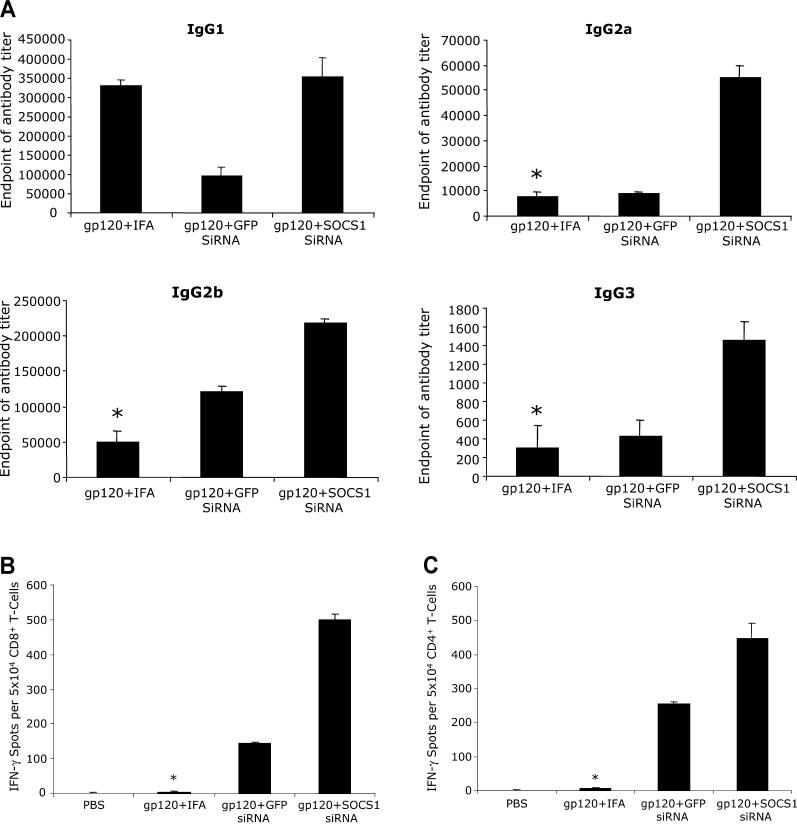
Comparison of gp120-Specific Antibody and T Cell Responses Induced by Protein Immunization and SOCS1-Silenced DCs Groups of C57BL/6 mice were immunized with gp120 protein (20 μg/ml)-pulsed, transduced BM-derived DCs (1 × 10^6^ cells/mouse) or the same amount of gp120 protein formulated in IFA (20 μg/mouse) twice at a weekly interval. All of the mice were injected with PolyI:C or R837 (30 μg/mouse) in vivo three times on days 1, 3, and 5 after each immunization, and sera and splenocytes were collected from each group of mice 14 d later. HIV gp120-specific IgG subclass titers (A) from the pooled sera of each group (4–6 mice/group) were quantified by capture ELISA. CD8^+^ T cells (B) and CD4^+^ T cells (C) isolated from pooled splenocytes were used for IFN-γ ELISPOT assays stimulated with gp120 proteins. Representative data from one of three experiments are presented. *P < 0.01, gp120 protein + IFA versus gp120-pulsed LV-SOCS1-siRNA-DCs.

We next asked whether SOCS1 silencing could enhance HIV Env-specific CTL responses by using IFN-γ ELISPOT, intracellular cytokine staining, and CTL assays to test the functional status of CD8^+^ T cells in the immunized mice. CTL activities against gp120-pulsed target cells in the LV-SOCS1-siRNA-DC mice were more potent than those in the LV-GFP-siRNA-DC mice (see Figure S1). The CTL activity detected in these assays was gp120-specific, since splenocytes from LV-SOCS1-siRNA-DC mice lacked any apparent CTL activity against non-gp120-pulsed target cells (unpublished data). Natural killer cell (NK) activities were also enhanced in mice immunized with SOCS1-silenced DCs (Figure S3). In vivo stimulation with PolyI:C or R837 further enhanced the CD8^+^ T cell responses in LV-SOCS1-siRNA-DC-immunized mice (Figure 1). Intracellular staining of splenocytes with IFN-γ also showed higher percentages of IFN-γ^+^ CD8^+^ T cells in LV-SOCS-siRNA-DC mice (Figure 1). Various TLR agonists had a comparable ability to enhance the immunostimulatory potency of LV-SOCS1-siRNA-DCs (Figure S4). In addition, the percentage of perforin-positive CD8^+^ T cells was significantly increased in mice immunized with gp120-pulsed LV-SOCS1-siRNA-DCs (Figure S5), suggesting that SOCS1-silenced DCs may qualitatively enhance CTL responses as well. LV-SOCS1-siRNA-DC immunization induced enhanced HIV gp120-specific CD4^+^ T cells (Figure 1). We compared the potency of SOCS1-silenced DC immunization and protein adjuvant immunization. Figure 2A–2C show that gp120-pulsed SOCS1-silenced DCs induced potent CD8^+^ and CD4^+^ T cell responses as well as antibody responses, especially IgG2a, IgG2b, and IgG3. In contrast, immunization with the same amount of recombinant gp120 proteins formulated in incomplete Freund's adjuvant (IFA) only induced weaker antibody responses and barely detectable CD8^+^ and CD4^+^ T cell responses. Taken together, these results demonstrate a balanced and enhanced antibody and T cell response against HIV Env in mice immunized with SOCS1-silenced DCs, especially when stimulated in vivo with a low dose of TLR agonists, suggesting that SOCS1 in DCs critically regulates both anti-HIV humoral and cellular immunity.

### Enhanced gp120-Specific Th Response Induced by SOCS1-Silenced DCs

**Figure 3:**
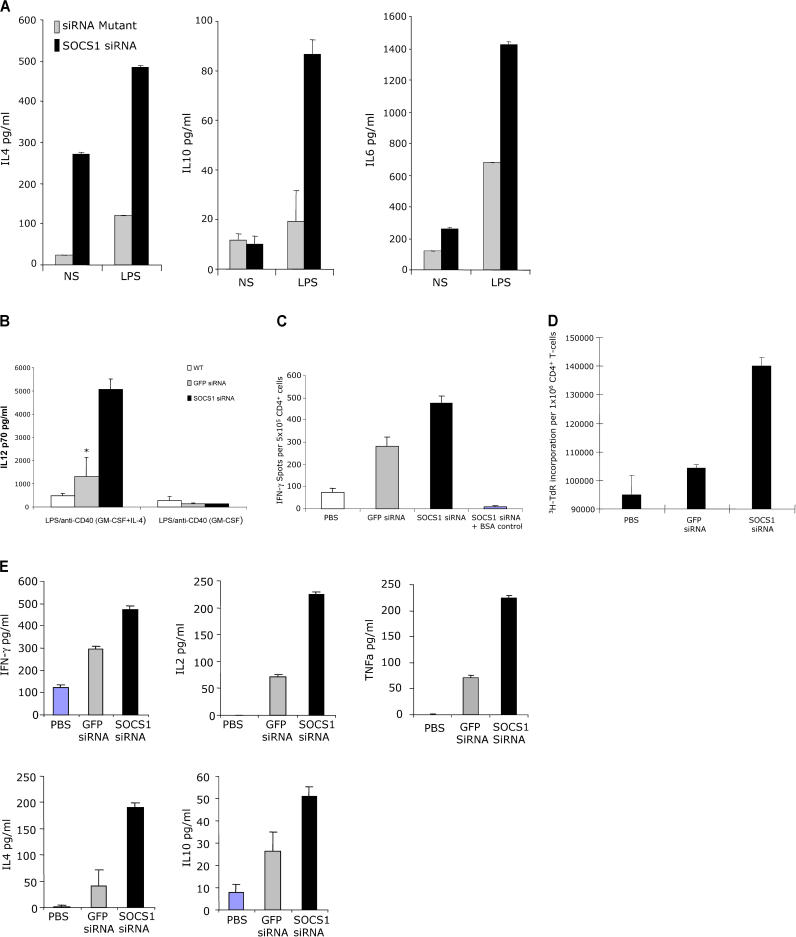
Enhanced Production of Both Th1- and Th2-Polarizing Cytokines by SOCS1-Silenced DCs and Activated CD4^+^ Th (A) Enhanced production of both Th1- and Th2-polarizing cytokines by SOCS1-silenced DCs. BM-DCs transfected with SOCS1 siRNA or control [24] were stimulated with LPS (100 ng/ml). Concentrations of various cytokines in the culture media were analyzed by ELISA 24 h after stimulation. NS, no stimulation. *P < 0.01, LV-SOCS1-siRNA-DCs versus LV-GFP-siRNA-DCs. (B) IL-12 production by transduced DCs. BM cells were cultured with mGM-CSF (20 μg/ml only or mGM-CSF and mIL-4 (20 μg/ml) [24] for 6 d and then transduced with LV-SOCS1-siRNA or LV-GFP-siRNA. The transduced DCs (5 × 10^5^/ml) were then stimulated with LPS (100 ng/ml) and plate-coated anti-CD40 mAb (5 μg/ml, BD Bioscience) in the presence or absence of IL-4. Concentrations of IL-12 (p70) in the culture media were analyzed by ELISA 24 h after stimulation and are presented from one of three independent experiments. *P < 0.01, LV-SOCS1-siRNA (GM-CSF + IL-4) versus LV-GFP-siRNA (GM-CSF + IL-4). (C–E) CD4^+^ Th responses induced by LV-SOCS1-siRNA-DCs. CD4^+^ T cells were isolated from pooled splenocytes of different groups of mice and subjected to the following assays. Numbers of IFN-γ-producing CD4^+^ T cell precursors were determined with the ELISPOT assay (C). ^3^H-thymidine incorporation rates of the isolated CD4^+^ T cells were determined on the fourth day of restimulation with gp120-pulsed DCs (D). Cytokine levels in the culture medium of isolated CD4^+^ cells stimulated with gp120-pulsed DCs for 48 h were determined by ELISA (E). The mean results (+ standard error) from one of three experiments are presented. *P < 0.01, LV-SOCS1-siRNA-DC versus LV-GFP-siRNA-DC mice.

Given the role of cytokines in programming Th1 versus Th2 responses [31,32], we reasoned that SOCS1 silencing might affect CTL and antibody responses by regulating the production of cytokines by DCs. Figure 3A and 3B shows significantly increased levels of IL-12 (p70), IFN-γ, and TNFα, which promote Th1-polarized responses, produced by LV-SOCS1-siRNA-BM-DCs generated in the culture containing GM-CSF and IL-4, compared with GFP-siRNA-DCs generated with GM-CSF and IL-4 culture after stimulation with LPS or LPS with anti-CD40 antibodies. The expression of EOMES mRNA, a transcription factor involved in the regulation of IFN-γ, was enhanced in LV-SOCS1-siRNA-DCs compared with LV-GFP-siRNA-DCs after stimulation with LPS (Figure S6), in agreement with a recent report by Hanada et al [25]. In addition, significant increases of IL-4, IL-6, and IL-10, which promote Th2-polarized responses, were also seen in the SOCS1-silenced BM-DCs (*P* < 0.01). Interestingly, we found that both LV-SOCS1-siRNA-DCs and LV-GFP-siRNA-DCs generated in a culture containing GM-CSF alone only produced low levels of IL-12 (p70), even when stimulated with LPS and anti-CD40 antibodies. These results suggest that IL-4 is a potent enhancer of IL-12 production, which is supported by an earlier finding [33]. Collectively, these data suggest that the higher levels of both Th1- and Th2-promoting cytokines produced by SOCS1-silenced DCs may account for the enhanced ability of SOCS1-silenced DCs to induce both HIV Env-specific CTL and antibody responses.

SOCS1 silencing in DCs clearly promoted antibody and CTL responses, but it was unclear whether HIV Env-specific CD4^+^ Th responses, which are intimately involved in the induction of antibody and CTL responses, are also enhanced by SOCS1 silencing. We therefore isolated CD4^+^ T cells from immunized mice using CD4^+^ microbeads and analyzed them with various assays. As depicted in Figure 3C, the frequencies of gp120-specific CD4^+^ T cells were significantly higher in LV-SOCS1-siRNA-DC mice than in LV-GFP-siRNA-DC mice. ^3^H-thymidine incorporation assays showed that the CD4^+^ T cells from LV-SOCS1-siRNA-DC mice proliferated more actively than those from LV-GFP-siRNA-DC mice in response to stimulation with gp120-pulsed DCs (Figure 3D). Analysis of the cytokine profiles produced by CD4^+^ T cells isolated from LV-SOCS1-siRNA-DC mice after stimulation with gp120-pulsed DCs revealed increased levels of both Th1-polarizing (IFN-γ, IL-12, and TNFα) and Th2-polarizing (IL-4 and IL-10) cytokines (Figure 3E). These results indicate that SOCS1-silenced DCs induce an enhanced Th1-polarized, but a mixed Th1 and Th2, response against HIV Env, which is consistent with the Th1-polarized gp120-specific IgG subclass profile (higher IgG2a) shown in Figures 1B and 2B.

### Enhanced gp120-Specific B Cell Activation by SOCS1-Silenced DCs

**Figure 4:**
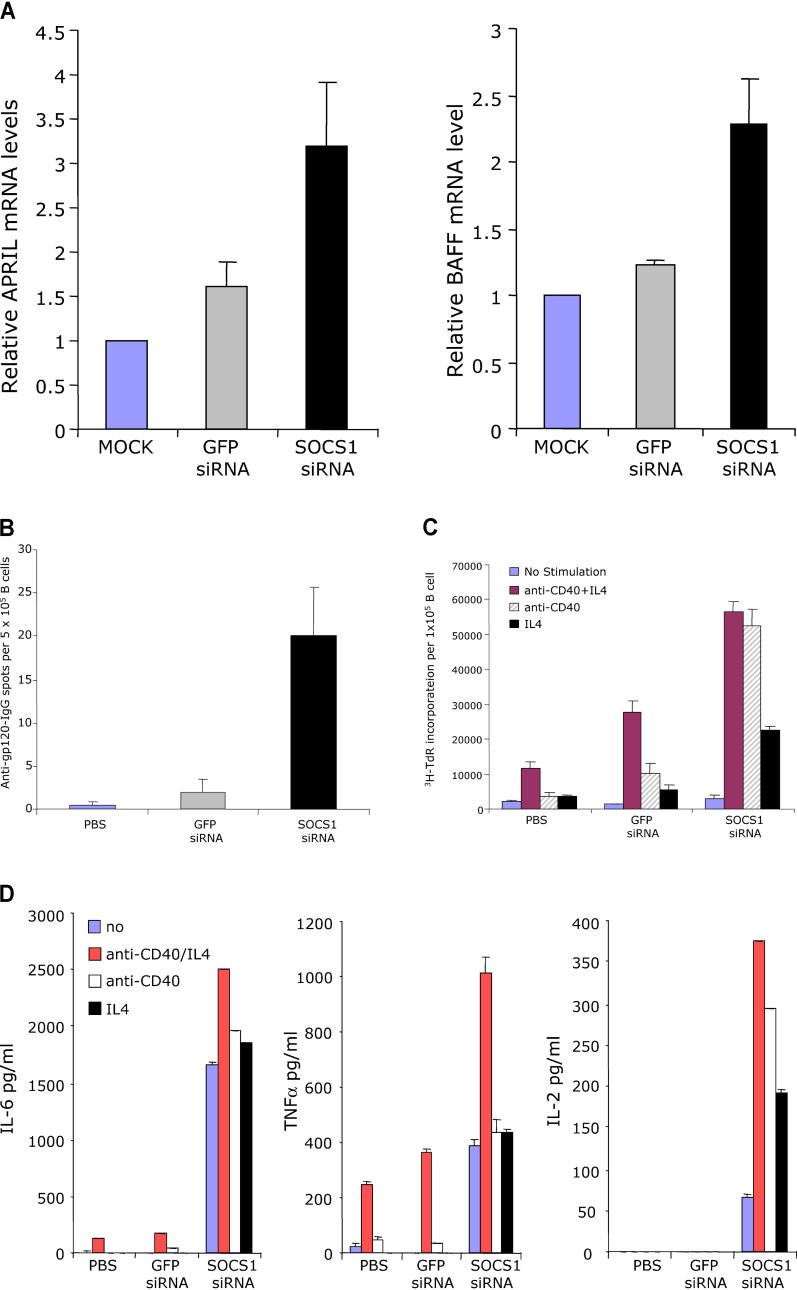
Enhanced HIV-Specific B Cell Responses (A) Enhanced production of BAFF and APRIL by SOCS1-silenced DCs. The transduced BM-DCs were stimulated with LPS (100 ng/ml) for 24 h. Relative expression levels of BAFF and APRIL mRNA were then determined by real-time quantitative PCR as described in Methods, and normalized to mock-transfected DCs after LPS stimulation using the Comparative Ct method [27]. Representative data from two independent experiments are presented. *P < 0.01, LV-SOCS1-siRNA-DC versus LV-GFP-siRNA-DCs. (B–D) Enhanced activation of gp120-specific B cells by SOCS1-silenced DCs. Frequencies of anti-gp120 antibody-producing cells in different groups of mice were determined and reported as the number of cells secreting gp120-specific IgG per 5 × 10^5^ B cells (B). The proliferation rates (C) and cytokine production (D) of B cells (5 × 10^4^/well) isolated from the spleens of different groups of mice after stimulation with anti-CD40 (5 μg/ml), IL-4 (20 ng/ml), or costimulation with anti-CD40 and IL-4 for 48 h were determined, and results from one of three independent experiments are presented. *P* < 0.01, LV-SOCS1-siRNA-DC mice versus LV-GFP-siRNA-DC mice under various in vitro stimulation conditions.

DCs have been shown to directly trigger B cell proliferation, maturation, and class-switch recombination by producing APRIL (a proliferation-inducing ligand) and BAFF (B-cell activating factor of the TNF family, also known as BLyS), members of the TNF superfamily [34–36]. We examined the effect of SOCS1 silencing on the production of APRIL and BAFF by DCs using real-time RT-PCR. LV-SOCS1-siRNA-DCs expressed higher levels of APRIL and BAFF mRNA upon LPS stimulation than did LV-GFP-siRNA-DCs (Figure 4A), in agreement with the increased expression of BAFF and APRIL in SOCS1^−/−^ DCs [20].

To test the ability of SOCS1-silenced DCs to enhance activation of gp120-specific B cells, we used an anti-gp120 IgG-specific B cell ELISPOT assay to directly examine the frequencies of anti-gp120 IgG-producing B cells in the immunized mice. Frequencies of anti-gp120 IgG-producing B cells were significantly higher in LV-SOCS1-siRNA-DC mice than in LV-GFP-siRNA-DC mice (*P* < 0.01) (Figure 4B). Higher percentages of B cells exhibited an activated phenotype characterized by high levels of CD69, CD40, and CD86 in LV-SOCS1-siRNA-DC mice, compared with B cells from LV-GFP-siRNA-DC mice (unpublished data). We further purified B cells from the spleens of immunized mice and stimulated them with various stimuli. Figure 4C shows that B cells from LV-SOCS1-siRNA-DC mice proliferated more vigorously when costimulated with anti-CD40 and IL-4 than did B cells from LV-GFP-siRNA-DC mice. Interestingly, B cells from LV-SOCS1-siRNA-DC mice, but not those from LV-GFP-siRNA-DC mice, responded strongly to IL-4 or anti-CD40 only, suggesting that increased numbers of B cells were already activated in vivo by immunization with LV-SOCS1-siRNA-DCs. We also found that B cells from LV-SOCS1-siRNA-DCs mice produced higher levels of various cytokines, including IL-6, IL-2, and TNF-α, in response to various stimuli (Figure 4D). The enhanced B cell activation and antibody production induced by SOCS1-silenced DCs were likely CD4^+^ T cell-dependent, since the antibody production was compromised in CD4 knockout mice immunized with SOCS1-silenced DCs (unpublished data). Collectively, these results suggest that SOCS1-silenced DCs produce enhanced levels of B lymphocyte stimulators (BAFF and APRIL) and Th2-polarizing cytokines, leading to more effective activation of HIV Env-specific B cells and Th cells.

### Long-Term HIV Env-Specific CTL and Antibody Responses Induced by SOCS1-Silenced DCs

**Figure 5:**
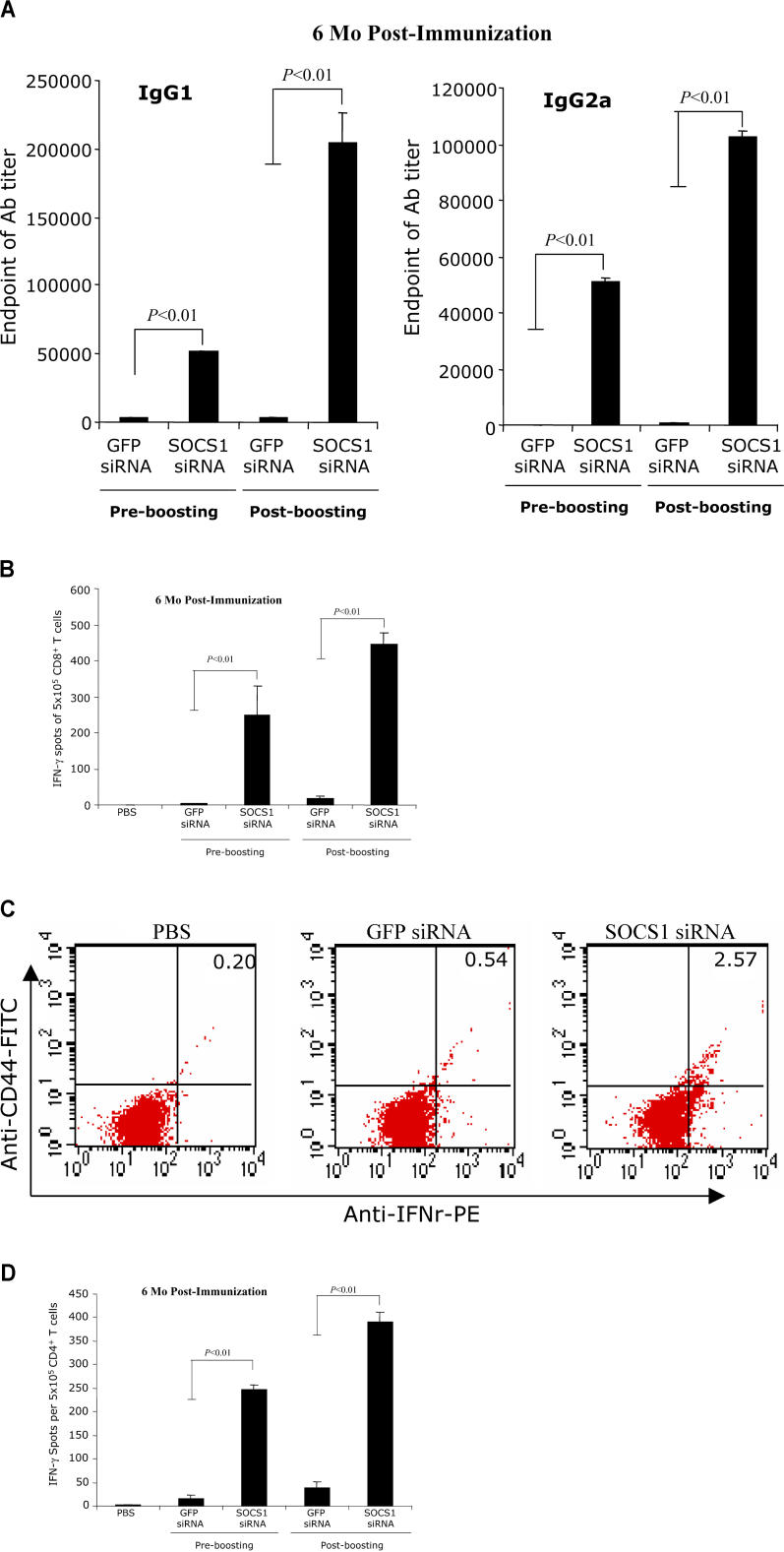
Long-Term gp120-Specific Antibody and CTL Responses Induced by SOCS1-Silenced DCs IgG subclass titers (A) from pooled sera of different groups of mice and frequencies of IFN-γ-positive T cells of CD8^+^ T cells (B) and CD4^+^ T cells (D) isolated from pooled splenocytes (two mice per group) were determined at 6 mo after DC immunization and on day 7 after booster immunization with recombinant gp120 emulsified in IFA (20 μg protein/mouse). Intracellular IFN-γ and surface CD44 costaining of gated CD8^+^ T cells from splenocytes at 6 mo after immunization are shown (C). The data are representative of two experiments.

Having shown that SOCS1 silencing in DCs enhances the primary HIV Env-specific CTL and antibody responses, we tested whether SOCS1-silenced DCs would induce memory HIV-specific CTL and antibody responses. Figure 5A shows that mice immunized with LV-GFP-siRNA-DCs had very low levels of gp120-specific antibodies at 6 mo after immunization, while LV-SOCS1-siRNA-DC mice still retained significant titers of gp120-specific IgG1 and IgG2 antibodies in their sera. At 1 wk after booster immunization, the LV-SOCS1-siRNA-DC mice showed strong recall antibody responses, with a mean titer of anti-gp120 IgG1 at 2 × 10^5^ and anti-gp120 IgG2 at 1 × 10^5^, while the LV-GFP-siRNA-DC mice showed poor recall antibody responses, with a mean titer of IgG1 at 3 × 10^3^ and IgG2 at 4 × 10^2^. These data show that SOCS1-silenced DCs exhibit about 64- and 255-fold increases in the titers of IgG1 and IgG2a antibodies, respectively, compared to LV-GFP-siRNA-DCs.

The maintenance of memory HIV-specific CTLs and Th was assessed by examining gp120-specific CD8^+^ and CD4^+^ T cell responses with IFN-γ ELISPOT assays. Figure 5B shows that strong gp120-specific CTL responses were detected in LV-SOCS1-siRNA-DC mice, but not in LV-GFP-siRNA-DC mice, at 6 mo after immunization (249 IFN-γ spots per 5 × 10^5^ CD8^+^ T cells in LV-SOCS1-siRNA-DC mice versus three IFN-γ spots in LV-GFP-siRNA-DC mice). Vigorous gp120-specific CTL responses were rapidly induced by booster immunization in LV-SOCS1-siRNA-DC mice, but not in LV-GFP-siRNA-DC mice (446 IFN-γ spots per 5 × 10^5^ CD8^+^ T cells in LV-SOCS1-siRNA-DC mice versus 16 IFN-γ spots in LV-GFP-siRNA-DC mice on day 7 post-boosting) (Figure 5B). Costaining of intracellular IFN-γ and the surface CD44 memory marker of CD8^+^ T cells also showed a higher percentage of CD44hi and IFN-γ^+^ CD8^+^ T cells in LV-SOCS1-siRNA-DC mice, compared with LV-GFP siRNA-DC mice at 6 mo post-immunization (Figure 5C). Similarly, gp120-specific CD4^+^ Th responses were maintained and rapidly induced in LV-SOCS1-siRNA-DCs mice at 6 mo after immunization (391 IFN-γ spots per 5 × 10^5^ CD4^+^ T-cells in LV-SOCS1-siRNA-DC mice versus 37 IFN-γ spots in LV-GFP-siRNA-DC mice on day 7 post-boosting) (Figure 5D). Thus, immunization with SOCS1-silenced DCs effectively induces long-term HIV Env-specific CTL, Th, and antibody responses.

No apparent toxicity was observed in the mice immunized with LV-SOCS1-siRNA-DCs pulsed with gp120 up to 7 mo after immunization. Histological analysis of all major organs and tissues of the immunized mice revealed no pathologic inflammation (unpublished data). Levels of IgG and anti-dsDNA were comparable in LV-SOCS1-siRNA-DC and mock DC mice. These data suggest that gp120-pulsed LV-SOCS1-siRNA-DC immunization does not cause pathological inflammation in mice.

### Resistance of SOCS1-Silenced DCs to HIV Env-Mediated Immune Suppression

**Figure 6:**
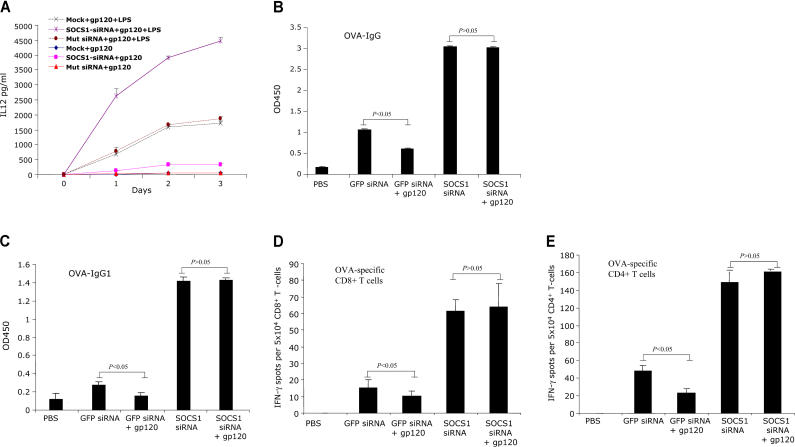
Resistance of SOCS1-Silenced DCs to HIV gp120-Mediated Suppression (A) Effects of gp120 on cytokine production by DCs. BM-DCs were transfected with SOCS1-siRNA or a SOCS1-siRNA mutant oligonucleotide as described previously [24], and then cultured with or without SF162 gp120 (20 μg/ml) or LPS (100 ng/ml), and cytokine levels were determined at the different times of cultures, as indicated. (B–E) Effects of gp120 on DC antigen presentation in vivo. Transfected BM-DCs were pulsed with OVA, incubated with or without gp120 for 2 d, and then stimulated with LPS (100 ng/ml) ex vivo overnight. Mice were then immunized with the transduced DCs twice, following three in vivo LPS stimulations. OVA-specific antibody IgG (B) and IgG1 (C) titers and frequencies of IFN-γ-producing OVA-specific CD8^+^ T cells (D) and CD4^+^ T cells (E) were examined 2 wk after the second DC immunization. Data are representative of two repeats.

The HIV Env protein gp120 can suppress the ability of DCs to produce proinflammatory cytokines and to stimulate T cells [37–40]. We therefore asked whether the enhanced activation of DCs by SOCS1 silencing might overcome the inhibitory effects of gp120 proteins on the cytokine production and immunostimulatory capacity of DCs. IL-12 was selected as a representative cytokine for these experiments, because DC-derived IL-12 was found to play a dual role, driving Th1 development as well as directly signaling B cells for developing humoral response [41–43]. As shown in Figure 6A, LV-SOCS1-siRNA-DCs in the presence of gp120 proteins retained the ability to respond to LPS. In contrast, the response of LV-GFP-siRNA-DCs to LPS stimulation was severely compromised by the presence of gp120 proteins. The susceptibility of SOCS1-silenced DCs to gp120-mediated suppression was further investigated in vivo. Mice were immunized with OVA-pulsed transduced DCs with or without pretreatment of gp120 proteins ex vivo. Pre-exposure to gp120 proteins did not have apparent effects on the ability of LV-SOCS1-siRNA-DCs to induce OVA-specific antibody responses (Figure 6B and 6C), nor did it compromise OVA-specific CD8^+^ CTL and CD4^+^ Th responses induced by LV-SOCS1-siRNA-DCs (*P* > 0.05) (Figure 6D and 6E). However, such pretreatment significantly reduced the ability of LV-GFP-siRNA-DCs to induce OVA-specific antibody and CTL responses (*P* < 0.05) (Figure 6B–6E). These results indicate that SOCS1 silencing renders DCs resistant to HIV gp120-mediated suppression, probably because of the enhanced cytokine production and hyperactivated state of SOCS1-silenced DCs [20,24].

### Regulation of gp120-Specific Cellular Responses by SOCS1-Restricted IL-12 Signaling in DCs

**Figure 7:**
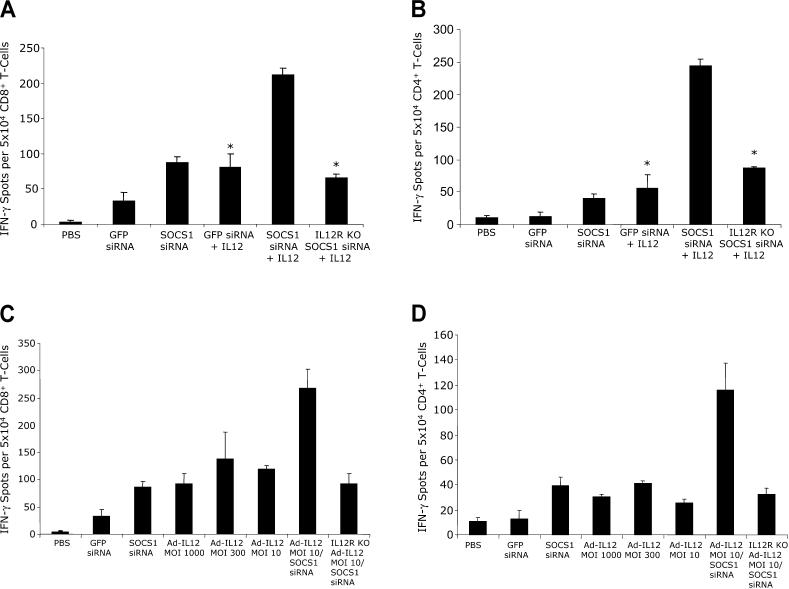
The Role of IL-12 Signaling in Enhanced Anti-HIV Immunity (A and B) In vivo injection with IL-12 preferentially enhanced gp120-specific CTL and Th responses induced by SOCS1-silenced DCs. C57BL/6 mice were immunized with 1 × 10^6^ of HIV gp120-pulsed (50 μg/ml), transduced DCs derived from BM of WT mice or IL-12 receptor KO mice with ex vivo TNFα maturation (50 ng/ml). On days 1, 3, and 5 after DC immunization, murine IL-12 (1 μg/mouse, Peprotech) was administered intraperitoneally. CD8^+^ T cells (A) or CD4^+^ T cells (B) isolated 2 wk later from the pooled splenocytes of immunized mice (2–3 each group) were subjected to IFN-γ ELISPOT assays. An irrelevant protein, OVA, was used as a negative control. Representative data from two independent experiments are presented. *P < 0.01, LV-SOCS1-siRNA-DC versus LV-SOCS1-siRNA-DC + IL-12, or IL12R KO LV-SOCS1-siRNA-DC + IL-12 versus LV-SOCS1-siRNA-DC + IL-12. (C and D) gp120-specific CTL and Th responses induced by SOCS1-silenced DCs or Ad-IL-12-DCs. BM-derived DCs from WT mice were transfected with LV-SOCS1-siRNA (MOI of 5) or Ad-IL-12 with various MOIs of 10–1,000 or cotransfected with LV-SOCS1-siRNA (MOI of 5) and Ad-IL-12 (MOI of 10) for 4 h. DCs derived from BM of IL-12 receptor KO mice were cotransfected with LV-SOCS1-siRNA (MOI of 5) and Ad-IL-12 (MOI of 10) for 4 h. Groups of C57BL/6 mice were immunized with 1 × 10^6^ of gp120-pulsed (50 μg/ml), transfected DCs with ex vivo TNFα maturation. CD8^+^ T-cells (C) or CD4^+^ T cells (D) isolated 2 wk later from the pooled splenocytes of immunized mice (2–3 each group) were subjected to IFN-γ ELISPOT assays. An irrelevant protein, OVA, was used as a negative control. Representative data from two independent experiments are presented. *P* < 0.01, Ad-IL-12/SOCS1-siRNA-DC versus IL-12-DCs, or Ad-IL-12/SOCS1-siRNA-DC versus IL12R KO Ad-IL-12/SOCS1-siRNA-DC.

Since IL-12 is a potent stimulator of Th1 immune responses [44] and is regulated by SOCS1 [45], we further tested whether in vivo stimulation with IL-12, which may be applicable in the clinic, is also effective in enhancing the potency of LV-SOCS1-siRNA-DCs. HIV gp120-pulsed, transduced DCs that were matured ex vivo with TNFα were transferred into mice. The recipient mice were then stimulated in vivo three times with a low dose of recombinant mouse IL-12 (1 μg/mouse/injection). Figure 7A shows that gp120-specific CD8^+^ CTL activities in LV-SOCS1-siRNA-DC-immunized mice were significantly enhanced by in vivo IL-12 stimulation, as demonstrated by IFN-γ ELISPOT assay. In contrast, in vivo administration with IL-12 only had a modest effect on CTL activities in LV-GFP-siRNA-DC-immunized mice. Figure 7B also shows that in vivo IL-12 stimulation preferentially enhanced gp120-specific CD4^+^ Th responses induced by LV-SOCS1-siRNA-DCs. These results suggest that in vivo administration with a representative proinflammatory cytokine (IL-12) preferentially enhances the immunostimulatory ability of SOCS1-silenced DCs.

The preferential enhancement of the immunostimulatory ability of SOCS1-silenced DCs by a low dose of IL-12 in vivo suggests that SOCS1-restricted cytokine signaling in antigen-presenting DCs is critical in regulating antigen presentation and anti-HIV immunity. To further investigate this possibility, we compared the immunostimulatory ability of DCs transfected with a recombinant adenovirus expressing mouse IL-12 cytokine (Ad-IL-12) or with LV-SOCS1-siRNA. Mice were immunized with gp120-pulsed DCs transfected with various MOIs of Ad-IL-12 (10 to 1,000), DCs transfected with LV-SOCS1-siRNA (MOI of 5), or DCs cotransfected with LV-SOCS1-siRNA (MOI of 5) and Ad-IL-12 (MOI of 10). DCs transfected with Ad-IL-12 at a MOI of 300 constitutively produced a high level of IL-12, comparable to that produced by LV-SOCS1-siRNA-DCs after stimulation with LPS (unpublished data). Figure 7C and 7D show that gp120-specific CTL and Th responses were enhanced in mice immunized with DCs transfected with Ad-IL-12 (MOIs of 10 to 1,000), when compared with control LV-GFP-siRNA-DC. Interestingly, DCs transfected with either low or high MOIs of Ad-IL-12 induced comparable levels of gp120-specific CD8^+^ and CD4^+^ T cell responses (Figure 7C and 7D), suggesting that gp120-specific immune responses cannot be simply boosted by administration of increasing doses of proinflammatory cytokines. However, SOCS1-silenced DCs cotransfected with a low MOI (10) of Ad-IL-12 induced significantly more potent gp120-specific CD8^+^ CTL and CD4^+^ Th responses than SOCS1-silenced DCs or WT DCs transfected with either low or high MOIs (10 to 1,000) of Ad-IL-12 (Figure 7C and 7D), supporting a critical role of SOCS1-restricted IL-12 signaling in DCs for the induction of anti-HIV cellular responses. To further determine the role of autocrine signaling of IL-12 in antigen-presenting DCs, we compared the CTL and Th responses induced by DCs derived from IL-12 receptor KO mice or WT mice. These DCs were cotransfected with LV-SOCS1-sRNA and Ad-IL-12 and then injected into WT mice. Ad-IL-12/SOCS1-siRNA-DCs derived from IL-12 receptor KO mice exhibited a significantly reduced ability to induce gp120-specific CTL responses, compared with Ad-IL-12/SOCS1-siRNA DCs from WT mice (Figure 7C and 7D). Similarly, in vivo stimulation with IL-12 showed a significantly reduced ability to induce gp120-specific CTL and Th responses induced by IL-12R KO LV-SOCS1-siRNA-DCs, compared with WT LV-SOCS1-siRNA-DCs (Figure 7A and 7B). Taken together, these results indicate that SOCS1-restricted, autocrine signaling of proinflammatory cytokines such as IL-12 in DCs, in addition to the SOCS1-restricted production of proinflammatory cytokines by DCs, plays a critical role in controlling anti-HIV immune responses.

### Potency of HIV DNA Vaccine Enhanced by Coimmunization with SOCS1-siRNA DNA

**Figure 8:**
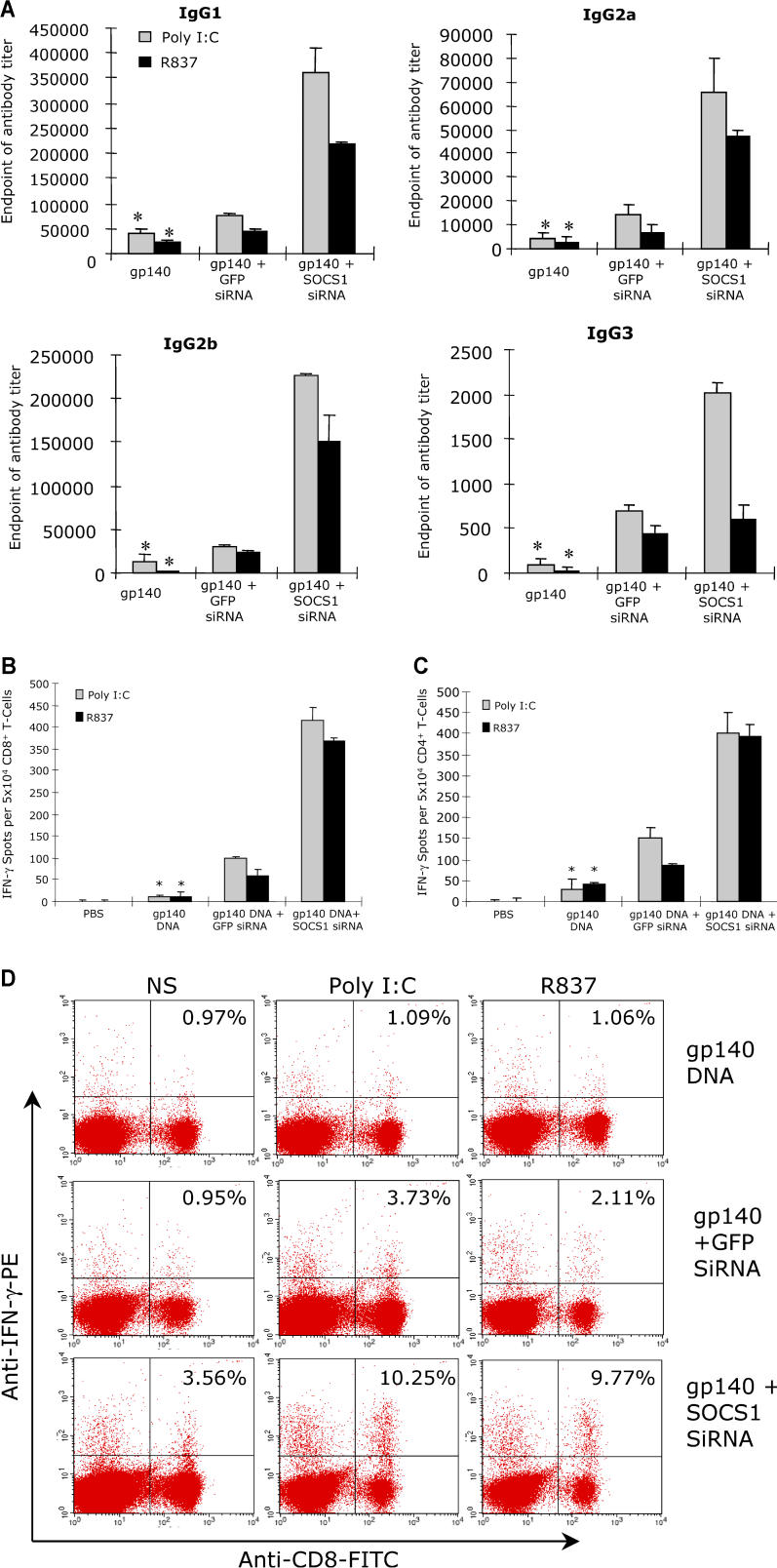
Enhancement of HIV DNA Vaccine by SOCS1-siRNA DNA Groups of C57BL/6 mice were immunized with gp140CF DNA [74] only or a mixture of gp140CF DNA and pSuper-SOCS1-siRNA expressor DNA [24] weekly for 3 wk, followed by PolyI:C or R837 stimulation (30 μg/mouse) in vivo three times on days 1, 3, and 5 after each DNA immunization. HIV gp120-specific IgG subclass titers (A), IFN-γ spot numbers of CD8^+^ T cells (B) and CD4^+^ T cells (C) from pooled splenocytes of different groups of mice (4–6 mice per group) 1 wk after the last immunization are shown from one of three independent experiments. Intracellular IFN-γ staining of CD8^+^ T cells from the pooled splenocytes was also performed (D). *P < 0.01, gp140CF and GFP siRNA versus gp140CF and SOCS1 siRNA coimmunization.

The ability of SOCS1-silenced DCs to enhance both HIV Env-specific CTL and antibody responses suggests that our SOCS1 silencing approach might be useful in improving the potency of HIV DNA vaccination. We generated a HIV gp140CF expression vector, in which the gp120/gp41 cleavage site and fusion domain of gp160 were deleted and the *gp140CF* gene was placed under control of the CMV promoter. To test the effect of SOCS1 siRNA on DNA vaccination, we injected mice with *gp140CF* DNA only or with a mixture of *gp140CF* DNA and pSuper-SOCS1-siRNA expressor DNA, which was constructed previously [24], weekly for 3 wk, followed by PolyI:C or R837 stimulation (30 μg/mouse) in vivo after each DNA immunization. Enhanced HIV Env-specific antibody titers were evident in mice coimmunized with pSuper-SOCS1-siRNA DNA (Figure 8). gp120-specific IgG2a antibodies, indicative of a Th1-polarized immune response [46], were preferentially enhanced, indicating that SOCS1-siRNA DNA preferentially enhances Th1-polarized anti-HIV immune responses induced by DNA vaccines. HIV Env-specific CTL responses were significantly enhanced by co-injection of pSuper-SOCS1-siRNA DNA, as demonstrated by ELISPOT assays (Figure 8). Intracellular IFN-γ staining also showed enhanced gp120-specific CD8^+^ T cell responses in mice coimmunized with pSuper-SOCS1 siRNA DNA (Figure 8). Moreover, HIV Env-specific CD4^+^ Th responses were enhanced by co-injection of SOCS1-siRNA DNA (Figure 8). These results indicate that pSuper-SOCS1 siRNA DNA coimmunization enhances the potency of HIV DNA vaccination, probably due to the enhanced immunostimulatory capacity of the cotransfected APCs in the immunized mice. Thus, our SOCS1 silencing strategy is applicable to ex vivo DC-based and in vivo vaccination settings.

## Discussion

In this study we found that silencing of the negative signaling regulator SOCS1 in DCs results in drastic enhancement of both HIV Env-specific CTL and antibody responses in mice. We demonstrated that SOCS1-silenced DCs have an enhanced ability to generate memory HIV Env-specific T cell and B cell responses. We also found that SOCS1-restricted, autocrine signaling of proinflammatory cytokines, such as IL-12 in DCs, as well as production of proinflammatory cytokines by DCs, play a critical role in inducing anti-HIV immune response. In addition, we demonstrated that coimmunization with SOCS1 siRNA DNA significantly enhances the potency of HIV DNA vaccination. Thus, a balanced memory humoral and cellular response against HIV can be induced by SOCS1-silenced DCs and SOCS1-siRNA DNA. This study indicates that SOCS1 functions as a critical antigen presentation attenuator. Furthermore, this SOCS1 silencing strategy is broadly applicable to enhancing both therapeutic and prophylactic vaccines against HIV and other pathogens.

The role of DCs in the induction of humoral responses has been traditionally viewed as a consequence of CD4^+^ Th priming for cognate interaction between T cells and B cells. However, the direct role of DCs in stimulation of the humoral response has been documented in vitro and in vivo [42,47]. Notably, DCs were found to strongly enhance both proliferation and antibody production of CD40-activated B cells [42]. Immunization with DCs loaded with antigens can induce a protective humoral response [48]. Here, we found that SOCS1-silenced DCs enhance the production of Th2-polarizing cyokines as well as B lymphocyte stimulatory cytokines (BAFF and APRIL), which is likely responsible for the enhanced Th and B cell activation seen in SOCS1-silenced DC-immunized mice. Our finding is supported by a previous report that SOCS1^−/−^ DCs induce aberrant expansion of B cells and autoreactive antibody production [20]. Hence, this study demonstrates the critical role of SOCS1 in DCs in controlling HIV-specific antibody responses and implies that the silencing of SOCS1 can be generically used to boost antibody responses against antigens other than HIV Env.

An important finding of this study is that SOCS1-silenced DCs induce balanced, memory HIV Env-specific antibody and CTL responses, which may be desirable for preventing or controlling HIV infection [5,8–11,49,50]. Although the mechanism(s) by which SOCS1 silencing induces a balanced, memory humoral and cellular response is unclear, it may involve the enhanced production of a mixed pattern of Th1- and Th2-polarizing cytokines by SOCS1-silenced DCs. These results are consistent with mixed antibody and CTL responses naturally generated against many pathogens such as viruses [30], indicating that Th1 and Th2 polarization is not mutually exclusive [32,51]. Hanada et al. recently found that the expression of Eomes, a transcription factor, was selectively overexpressed in SOCS1^−/−^ BM-DCs, which may be responsible for the enhanced production of IFN-γ [25]. Baetz et al. [22] and Gingras et al. [23] recently reported that SOCS1 indirectly regulates TLR signaling in macrophages by inhibiting the signaling of type I IFN that is induced by TLR signaling. However, the Gingras et al. studies [15,22,23] showed that levels of IL-12 produced by SOCS1^−/−^ BM-derived macrophages were comparable to those by WT BM-derived macrophages in response to LPS. We found that SOCS1-silenced BM-DCs produced higher levels of proinflammatory cytokines including IL-12 (p70 heterodimer) than did LV-GFP-siRNA-DCs (see Figure 2B). Our results are also consistent with two recent reports that SOCS1 KO macrophages produced excessive amounts of IL-12 and other cytokines in response to stimuli, and that enhanced levels of IL-12 and other cytokines were found in sera of conditional SOCS1 KO mice [52,53]. We noticed that transduction with control GFP-siRNA also enhanced the immunostimulatory potency of DCs, because siRNA molecules can stimulate DCs via the activation of TLR signaling [54,55] and the direct activation of some cellular genes such as the IFN-stimulated genes [56]. Thus, the enhanced immunostimulatory potency of DCs by SOCS1-siRNA is likely a collective result of SOCS1 silencing and nonspecific stimulatory effect of siRNA molecules. The nonspecific stimulatory ability of siRNA molecules may be an added benefit of using siRNA to silence a signaling inhibitor to enhance anti-HIV immunity.

The results of this study underscore the importance of autocrine SOCS1-restricted signaling of proinflammatory cytokines such as IL-12 in DCs for the induction of anti-HIV immune responses. Proinflammatory cytokines have been used to enhance the potency of HIV vaccines [57,58]. Here we found that in vivo stimulation with IL-12 or cotransfection of recombinant Ad-IL-12 viruses only modestly enhance anti-HIV immune responses induced by WT DC immunization. In contrast, the anti-HIV immune responses induced by SOCS1-silenced DC immunization were significantly enhanced by in vivo stimulation with IL-12 or cotransfection of Ad-IL-12. These results indicate that the signaling of proinflammatory cytokines and their stimulatory effects on APCs are tightly restricted by SOCS1, which may reflect the modest or no enhancement of the potency of HIV vaccines by coexpression of various proinflammatory cytokines [59]. The importance of autocrine signaling of proinflammatory cytokines in antigen presentation was reported in several recent studies [60,61]. In addition, we found that SOCS1-silenced DCs have a superior ability to generate HIV-specific memory T cell and B cell responses. The increase of memory T cell and antibody responses was more profound than that of primary responses induced by SOCS1-silenced DCs. Although the molecular mechanisms for memory generation are still poorly defined, the fate of activated T lymphocytes is likely determined by the strength under which an antigen is presented by APCs [62–66]. The results of our study suggest that unrestricted autocrine signaling, as well as enhanced production of both Th1- and Th2-promoting cytokines by SOCS1-silenced DCs, may account for the enhanced ability of SOCS1-silenced DCs to induce memory HIV-specific CTL and antibody responses. It is tempting to postulate that enhanced expression and signaling of cytokines such as IL-7 and IL-15 and other surface molecules, which are involved in the generation of memory response [67–69], by SOCS1-silenced DCs may be responsible for the enhanced memory responses. Our results also suggest that the quality of gp120-specific T cells, in addition to their frequencies, may be enhanced by SOCS1-silenced DCs (Figure S6). It would be interesting to systematically examine the expression of granzyme, perforin, Fas ligand, and other molecules on these antigen-specific T cells induced by SOCS1-silenced DCs in a future study.

Functional defects and depletion of DCs are common in HIV-infected individuals, likely contributing to the progressive immunodeficiency. Peripheral mononuclear cells from HIV-1-infected patients have been shown to produce significantly less IL-12 than those from uninfected controls [70]. HIV gp120 protein can suppress the ability of DCs to produce proinflammatory cytokines and to stimulate T cells [37,71], although the mechanism by which gp120 suppresses the production of proinflammatory cytokines is largely uncharacterized. We found that SOCS1-silenced DCs are more resistant to HIV gp120-mediated suppression, since SOCS1-silenced DCs in the presence of gp120 proteins still produced higher levels of IL-12 in response to LPS and induced stronger CTL and antibody responses (see Figure 6). This finding is especially relevant to the development of therapeutic HIV vaccines, which would be used in immunosuppressed HIV-infected individuals [72]. The enhanced resistance to gp120-mediated suppression may be due to the unbridled proinflammatory STAT (1/2/4) signaling in SOCS1-silenced DCs to antagonize anti-inflammatory signaling mediated by HIV gp120. In this study we treated DCs with high concentrations of soluble recombinant HIV gp120 proteins largely in a monomeric form, which may not reflect the physiologic conditions of HIV infection. Further studies are needed to investigate precise molecular mechanisms responsible for the enhanced resistance of SOCS1-silenced DCs to HIV suppression in a condition closely resembling natural HIV infection.

The vaccination strategy described here, to our knowledge, represents the first effort to enhance anti-HIV immune responses by inhibiting the host's immune inhibitors in DCs. Since natural immunity is ineffective in controlling HIV-1 infection, disabling the host's immune inhibitors may be critical to generate effective anti-HIV immune responses. However, mere enhancement of HIV-specific immune responses may not lead to the induction of protective HIV antibodies and CTL responses. In this regard, our approach offers the opportunity for combinational immunization with currently available vaccines, as demonstrated by our coimmunization of DNA vaccine and SOCS1 siRNA DNA. When used with improved HIV immunogens and delivery systems [5,73], this vaccination approach may provide a new avenue to enhance weak protective immune responses or generate broader and stronger responses not only against dominant epitopes, but also against weakly immunogenic or cryptic, yet protective epitopes.

Current efforts in HIV immunotherapy and vaccine development are largely aimed at stimulating anti-HIV immune responses by modifying HIV antigens and using various delivery systems and adjuvants. This study demonstrates the principle of disabling a signaling inhibitor in host DCs as an alternative and effective HIV immunization approach, which could be translated into the clinic. Indeed, our recent study identified a human SOCS1 siRNA and demonstrated that human SOCS1-silenced DCs also had enhanced immunostimulatory capacity to activate antigen-specific CTL responses (unpublished data). The SOCS1 siRNA molecule or expression cassette can be incorporated into various forms of prophylactic HIV vaccines, including DNA-, adenovirus-, vaccinia-, poxvirus-, virus-like particle-, and other vector-based vaccines. Furthermore, immunization of HIV-infected patients with DCs loaded with inactivated HIV viruses led to a reduction in viral loads and an increase in CD4^+^ T cell numbers in the blood [72], suggesting that SOCS1 silencing could augment the effect of therapeutic HIV vaccines for the long-term control of HIV infection. Thus, further investigations are warranted to determine if protective anti-HIV responses would be induced by this SOCS1 silencing strategy in monkeys and ultimately in humans.

## Supporting Information

Figure S1 Enhanced gp120-Specific Antibody and CTL Responses Induced by SOCS1-Silenced DCs Groups of C57BL/6 mice were immunized with gp120 (SF162) protein-pulsed, transduced BM-derived DCs (1 × 10^6^ cells/mouse) twice at a weekly interval without in vivo LPS stimulation, and sera and splenocytes were collected from each group of mice 14 d later. HIV gp120-specific IgG (A) and subclass (B) titers from the pooled sera of each group (4–6 mice/group) were quantified by capture ELISA. Antibody titers are reported as the mean ± standard deviation of endpoint titers [24]. Pooled splenocytes from the immunized mice were subjected to CTL assays against gp120 protein-pulsed syngeneic TC-1 cells (C). Representative data from one of three experiments are presented. *P < 0.01, LV-SOCS1-siRNA-DCs versus LV-GFP-siRNA-DCs.
(74 KB PDF).

Figure S2 gp120-Specific Antibody and CTL Responses Enhanced by SOCS1-Silenced DCs and In Vivo LPS Stimulation Groups of C57BL/6 mice were immunized with gp120 (SF162) protein-pulsed, transduced BM-derived DCs (1 × 10^6^ cells/mouse) twice at a weekly interval, followed by LPS stimulation (30 μg/mouse) in vivo three times on days 1, 3, and 5 after each DC immunization, and sera and splenocytes were collected from each group of mice 14 d later. HIV gp120-specific IgM and IgG (A) and IgG subclass (B) titers from the pooled sera of each group (4–6 mice/group) were quantified by capture ELISA. Pooled splenocytes from the immunized mice were subjected to CTL assays against gp120 protein-pulsed syngeneic TC-1 cells (C). CD8^+^ T cells isolated from the splenocytes were used for IFN-γ ELISPOT assays stimulated with gp120 proteins or control BSA (D). Representative data from one of three experiments are presented. *P < 0.01, LV-SOCS1-siRNA-DCs versus LV-GFP-siRNA-DCs.
(72 KB PDF).

Figure S3 Enhanced NK Activities in Mice Immunized with SOCS1-Silenced DCs Splenocytes pooled from each group of mice immunized with gp120-pulsed BM-DCs (1 × 10^6^ cells/mouse) were examined for NK activity using a 5-h ^51^Cr release assay against Yac-1 cells. Data are representative of three independent experiments. *P* < 0.05, LV-SOCS1-siRNA-DCs versus LV-GFP-siRNA-DCs.
(50 KB PDF).

Figure S4 Comparison of T Cell Responses Boosted by Various TLR Agonists Groups of C57BL/6 mice were immunized with gp120 protein-pulsed, LV-SOCS1-siRNA-transduced DCs (1 × 10^6^ cells/mouse) twice at a weekly interval, followed by in vivo stimulation with LPS, PolyI:C, or R837 (30 μg/mouse) three times on days 1, 3, and 5 after each DC immunization. CD8^+^ T cells isolated from the splenocytes were used for IFN-γ ELISPOT assays stimulated with gp120 proteins 14 d later. NS, no in vivo stimulation with any TLR agonist. *P < 0.01, NS versus LPS, PolyI:C, or R837.
(48 KB PDF).

Figure S5 Enhanced Perforin Expression in T Cells of Mice Immunized with SOCS1-Silenced DCs Groups of C57BL/6 mice were immunized with gp120-pulsed, transduced BM-derived DCs (1 × 10^6^ cells/mouse) or the same amount of gp120 protein formulated in IFA (20 μg/mouse) twice at a weekly interval. All of the mice were injected with PolyI:C (30 μg/mouse) in vivo three times on days 1, 3, and 5 after each immunization, and splenocytes were collected from each group of mice 14 d later. The splenocytes were in vitro restimulated with gp120 protein-pulsed BM-DCs for 5 h and then costained with anti-CD8-FITC and anti-Perforin-PE (BD Pharmingen) for FACS analysis.
(66 KB PDF).

Figure S6 Enhanced Expression of Eomes in LV-SOCS1-siRNA-Transduced DCs The expression of Eomes and T-bet in the transduced DCs after 24 h of LPS stimulation was examined by RT-PCR, as described by Hanada et al. [25]. GAPDH was used as an internal control. A pair of primers used for T-bet amplification were 5′- CCCACAAGCCATTACAGG-3′ and 5′- AGTGATCTCTGCGTTCTGGT-3′; a pair of primers for Eomes amplification was 5′- TGAATGAACCTTCCAAGACTCAGA-3′ and 5′- GGCTTGAGGCAAAGTGTTGACA-3′; and a pair of primers for GAPDH amplification was 5′- ACCACAGTCCATGCCATCAC-3′ and 5′- TCCACCACCCTGTTGCTGTA-3′.
(75 KB PDF).

**Patient Summary**

**Background**

When you are vaccinated against a virus, you are given a harmless form of the virus. A particular part of the virus—a part called an “antigen”—triggers your immune system to produce a response that includes antibodies (proteins secreted into the blood that bind just to that antigen) and specific cells. If one day you are infected with the actual virus, your body now has the means to fight it off. Despite a great deal of work going into the development of a vaccine against HIV, so far no vaccine has been produced. There are a number of possible reasons for this lack of success. For example, it has not been easy to identify the ideal part on the virus (the ideal antigen) that would trigger the immune system to produce a response. Also, we have not yet found a way to trigger a strong enough immune response against any experimental HIV vaccine. One group of cells involved in producing an immune response is known as antigen-presenting cells. These cells are responsible for handling antigens from viruses (and other microbes, such as bacteria) so that the body's immune system can respond efficiently and eliminate the microbe. The activity of these cells is controlled by molecular signals (cytokines) inside the cell.

**Why Was This Study Done?**

The authors wanted to take a different approach from the one described above to producing a vaccine. Our immune systems are kept in check by molecules called “immune inhibitors.” These prevent our immune systems from attacking our own bodies. These inhibitors might also be preventing our own immune systems from mounting an effective immune response to an HIV vaccine. The authors thought that by “switching off” or silencing these inhibitors, this might help the immune system to trigger a response against an HIV vaccine. They tested their hypothesis in a laboratory study of mice. They switched off a particular immune inhibitor called “SOCS1” inside a type of antigen-presenting cell called a dendritic cell, and looked at whether this would boost the mouse immune response in general, and whether it would lead to a more effective immune response against an HIV vaccine.

**What Did the Researchers Do and Find?**

They found that silencing SOCS1 in dendritic cells increased the immune response, and that this response was long-lasting. They also showed that when this strategy was combined with vaccination using genetic material (DNA) from HIV, the vaccine was more effective.

**What Do These Findings Mean?**

It seems that it may be possible to boost the body's response to HIV vaccination by switching off an immune inhibitor. However, these results are preliminary, as this approach has at the moment not been tested in humans, and before it is clear whether it will work in humans much research will need to be done, including assessing whether the approach is safe.

**Where Can I Get More Information Online?**

Medline Plus has many links to pages of information on HIV:

http://www.nlm.nih.gov/medlineplus/aids.html

The Body has a page of links on the search for an HIV vaccine:

http://www.thebody.com/treat/vaccines.html

## Abbreviations

APC: antigen-presenting cell
BM: bone marrow
CTL: CD8^+^ cytotoxic T lymphocyte
DC: dendritic cell
ELISPOT: enzyme-linked immunospot
Env: envelope
GM-CSF: granulocyte-macrophage colony-stimulating factor
HIV: human immunodeficiency virus
IFA: incomplete Freund's adjuvant
IFN: interferon
IL: interleukin
JAK: Janus kinase
KO: knockout
LPS: lipopolysaccharide
LV: lentiviral vector
MOI: multiplicity of infection
NK: natural killer cell
OVA: ovalbumin
siRNA: small interfering RNA
SOCS: suppressor of cytokine signaling
STAT: signal transducer and activator of transcription
Th: CD4^+^ T helper cell
TLR: Toll-like receptor
WT: wild-type
