# Supplementary material for: An Alternative and Effective HIV Vaccination Approach Based on Inhibition of Antigen Presentation Attenuators in Dendritic Cells
Source: PLoS Med. 2006 Jan 3;3(1):e11. doi: 10.1371/journal.pmed.0030011 (PMC1323501; doi:10.1371/journal.pmed.0030011)
Supplement: Supplementary file 1 [file pmed.0030011.sg001.pdf]

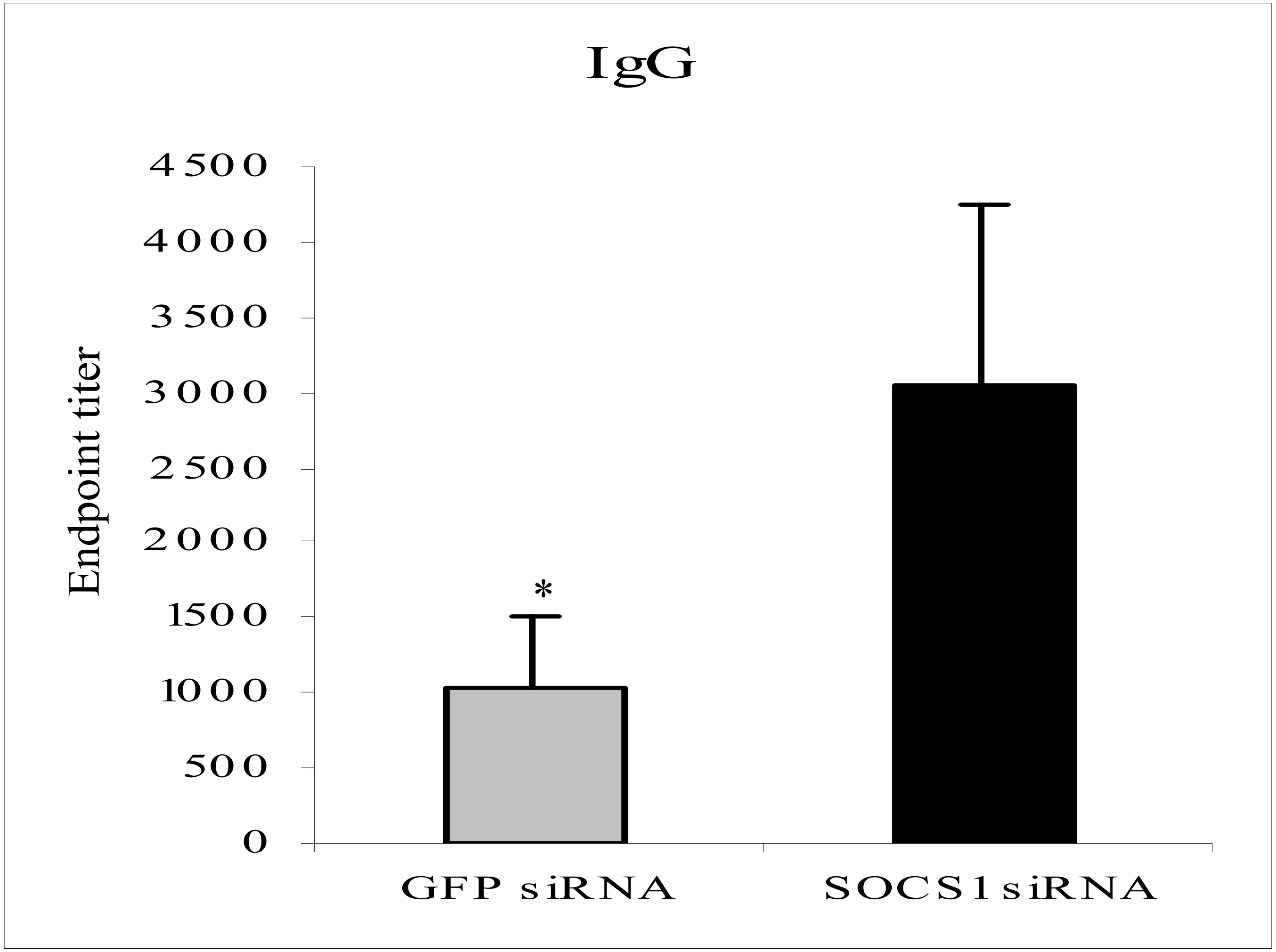
IgG
Endpoint titer
4500
4000
3500
3000
2500
2000
1500
1000
500
0
*
GFP siRNA
SOCS1siRNA

S-Fig. 1A

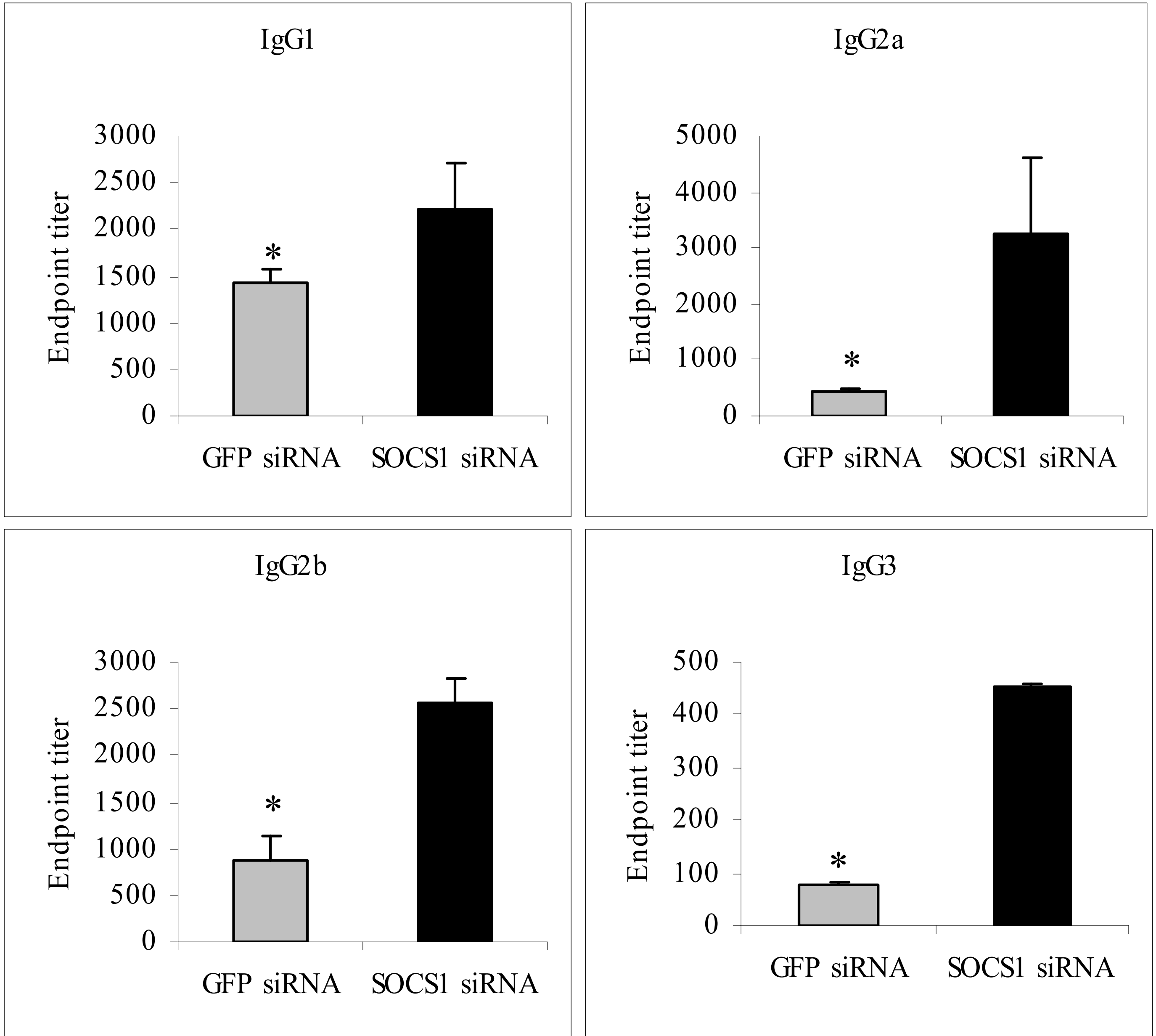
IgG1
3000
2500
2000
1500
1000
500
0
Endpoint titer
*
GFP siRNA
SOCS1 siRNA
IgG2a
5000
4000
3000
2000
1000
0
Endpoint titer
*
GFP siRNA
SOCS1 siRNA
IgG2b
3000
2500
2000
1500
1000
500
0
Endpoint titer
*
GFP siRNA
SOCS1 siRNA
IgG3
500
400
300
200
100
0
Endpoint titer
*
GFP siRNA
SOCS1 siRNA

S-Fig. 1B

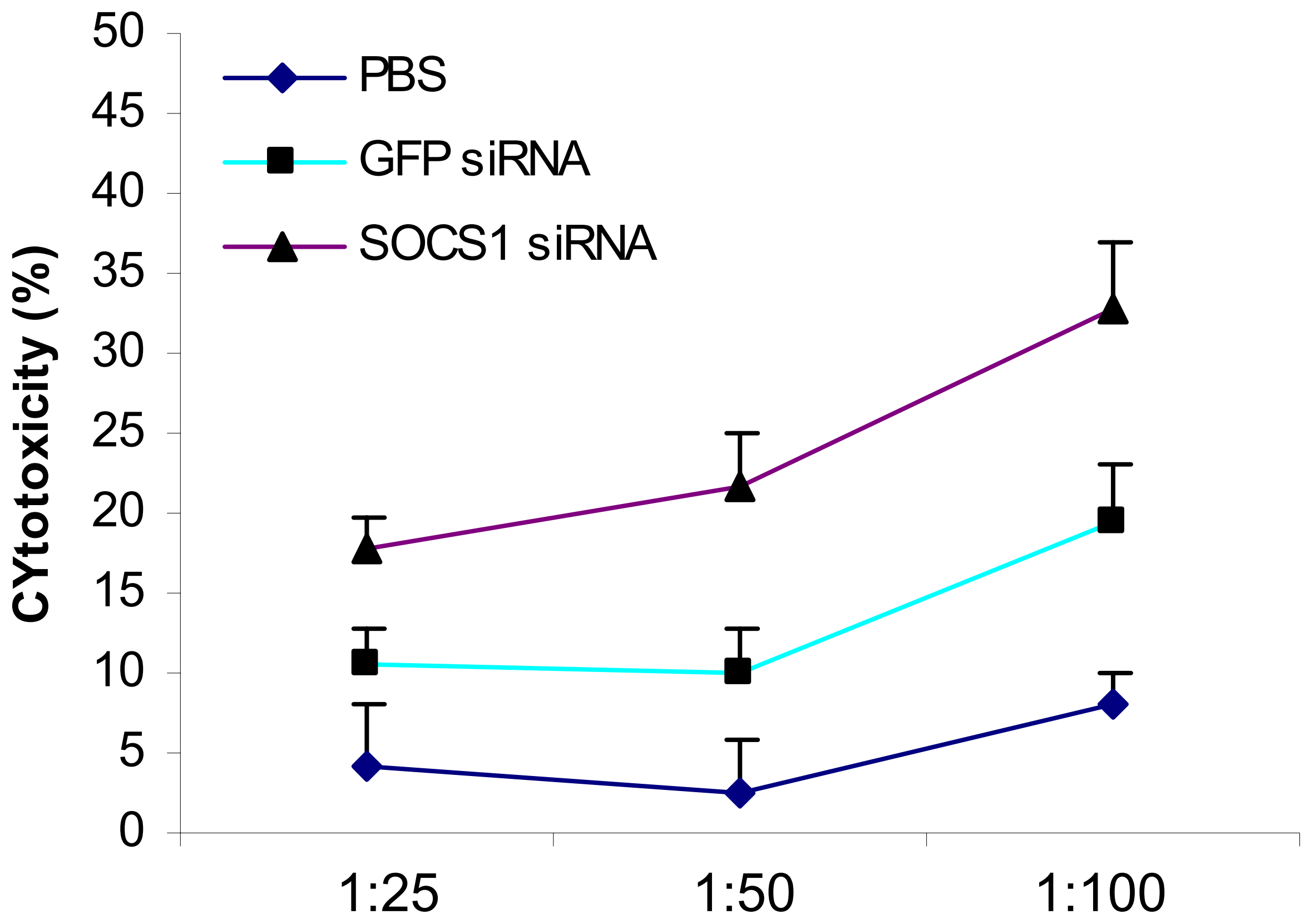

CYtotoxicity (%)
50
45
40
35
30
25
20
15
10
5
0
PBS
GFP siRNA
SOCS1 siRNA
1:25
1:50
1:100

S-Fig. 1C
