# Supplementary material for: An Alternative and Effective HIV Vaccination Approach Based on Inhibition of Antigen Presentation Attenuators in Dendritic Cells
Source: PLoS Med. 2006 Jan 3;3(1):e11. doi: 10.1371/journal.pmed.0030011 (PMC1323501; doi:10.1371/journal.pmed.0030011)
Supplement: Supplementary file 2 [file pmed.0030011.sg002.pdf]

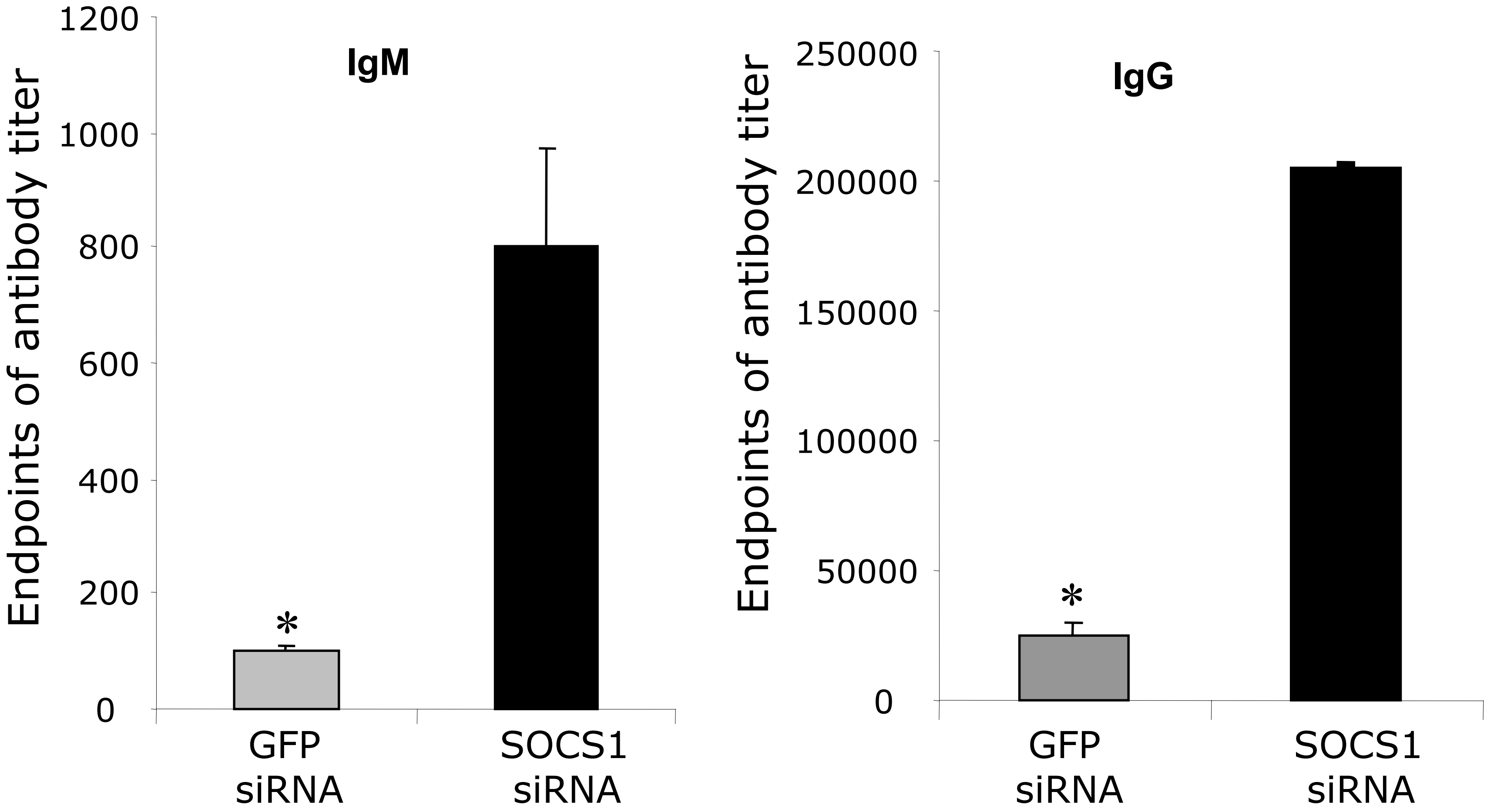
IgM
Endpoints of antibody titer
1200
1000
800
600
400
200
0
*
GFP siRNA
SOCS1 siRNA
IgG
Endpoints of antibody titer
250000
200000
150000
100000
50000
0
*
GFP siRNA
SOCS1 siRNA

S- Fig. 2A

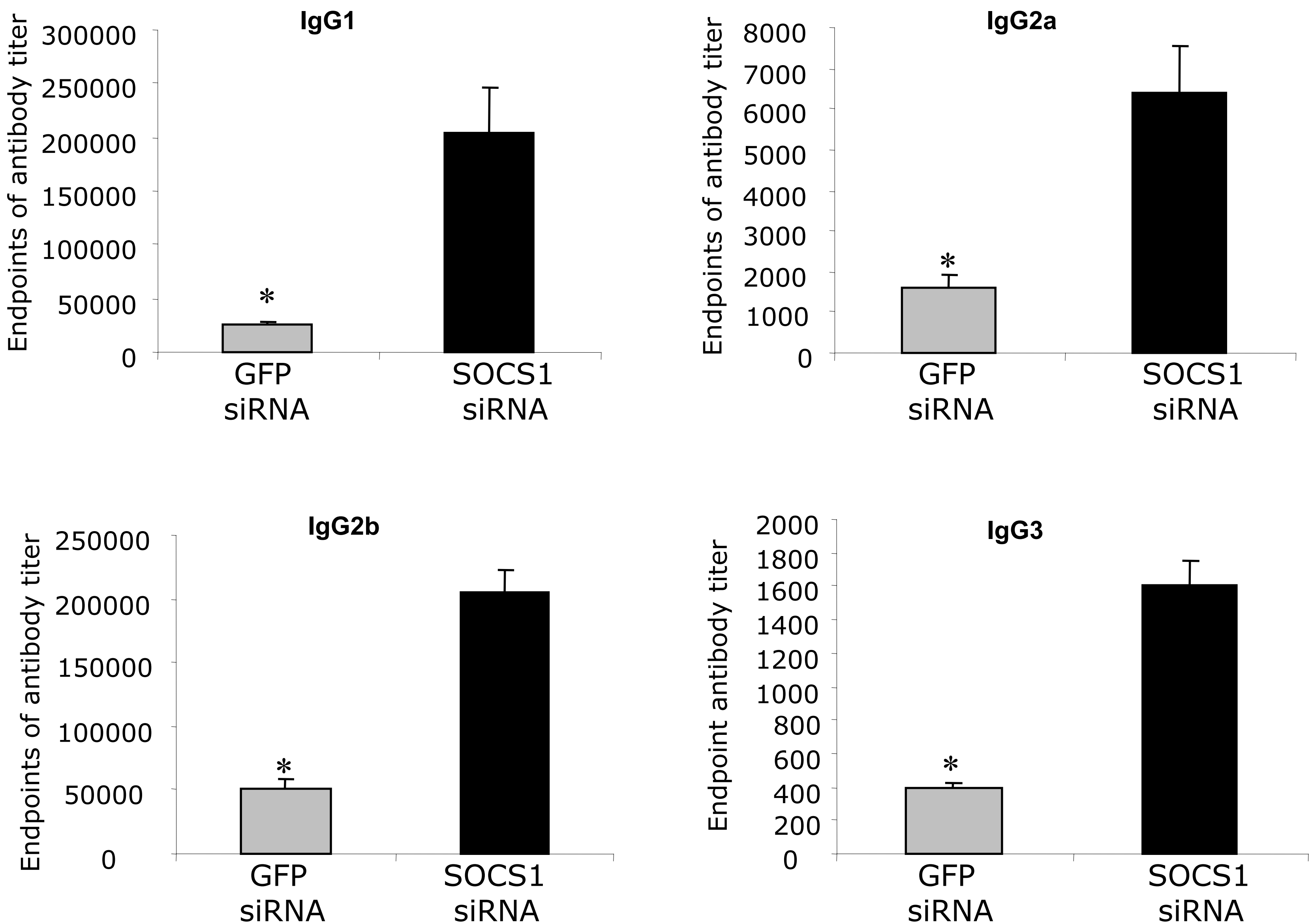

IgG1
Endpoints of antibody titer
300000
250000
200000
150000
100000
50000
0
GFP siRNA
SOCS1 siRNA
IgG2a
Endpoints of antibody titer
8000
7000
6000
5000
4000
3000
2000
1000
0
GFP siRNA
SOCS1 siRNA
IgG2b
Endpoints of antibody titer
250000
200000
150000
100000
50000
0
GFP siRNA
SOCS1 siRNA
IgG3
Endpoint antibody titer
2000
1800
1600
1400
1200
1000
800
600
400
200
0
GFP siRNA
SOCS1 siRNA

S- Fig. 2B

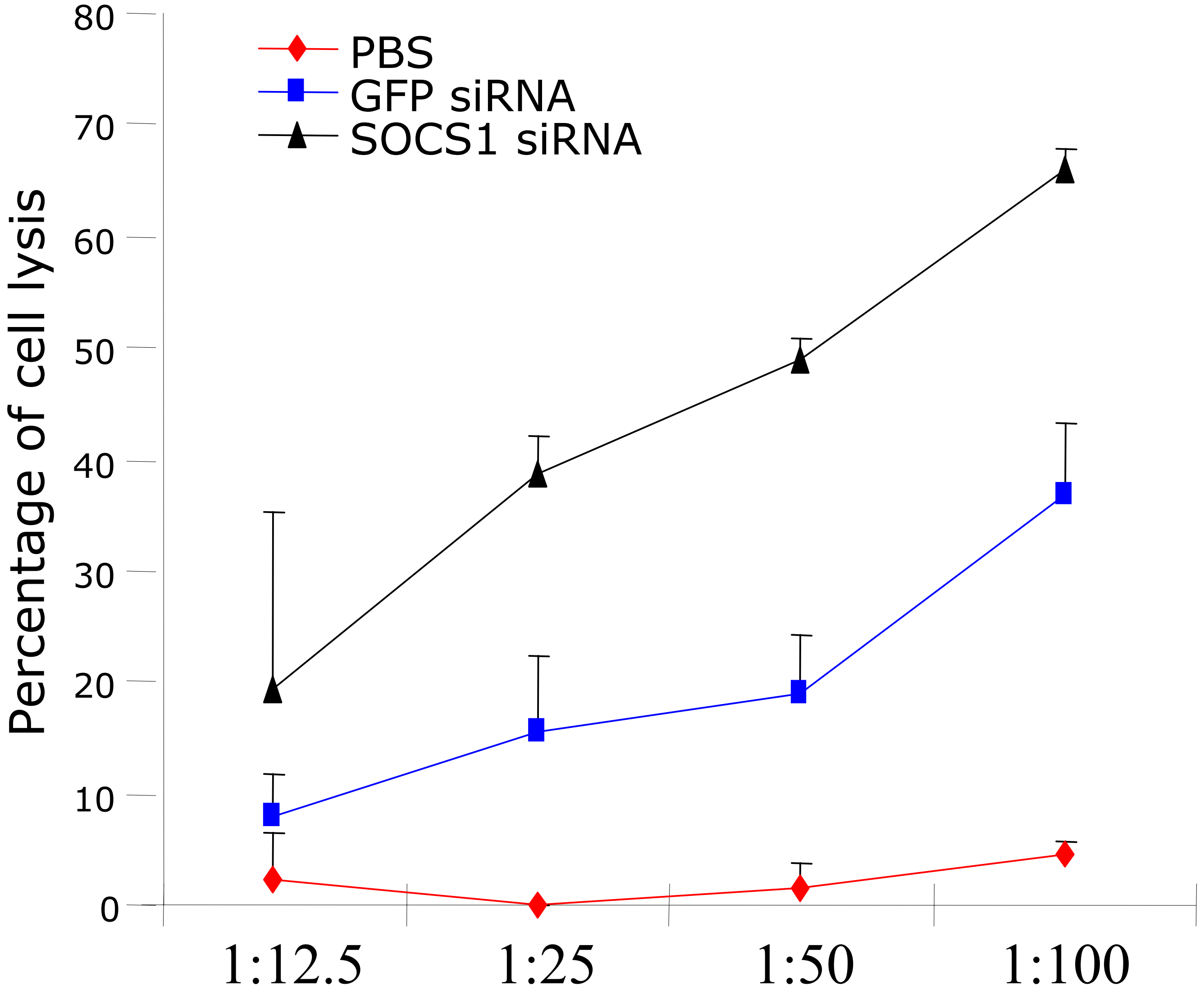
PBS
GFP siRNA
SOCS1 siRNA
Percentage of cell lysis
0
10
20
30
40
50
60
70
80
1:12.5
1:25
1:50
1:100

S-Fig. 2C

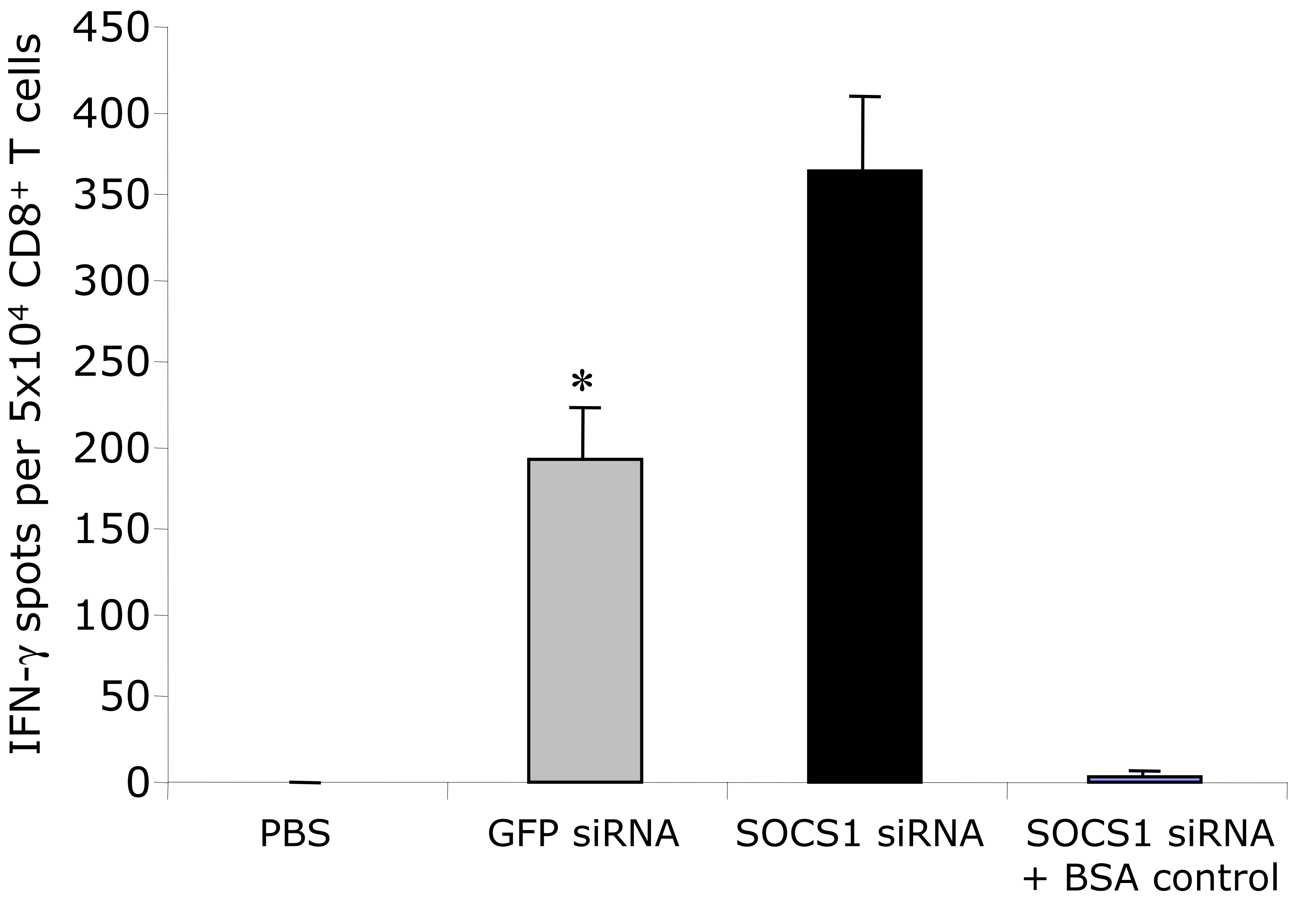

IFN-γ spots per 5x10⁴ CD8⁺ T cells
450
400
350
300
250
200
150
100
50
0
*
PBS
GFP siRNA
SOCS1 siRNA
SOCS1 siRNA
+ BSA control

S-Fig. 2D
