# Supplementary material for: An Alternative and Effective HIV Vaccination Approach Based on Inhibition of Antigen Presentation Attenuators in Dendritic Cells
Source: PLoS Med. 2006 Jan 3;3(1):e11. doi: 10.1371/journal.pmed.0030011 (PMC1323501; doi:10.1371/journal.pmed.0030011)
Supplement: Supplementary file 3 [file pmed.0030011.sg003.pdf]

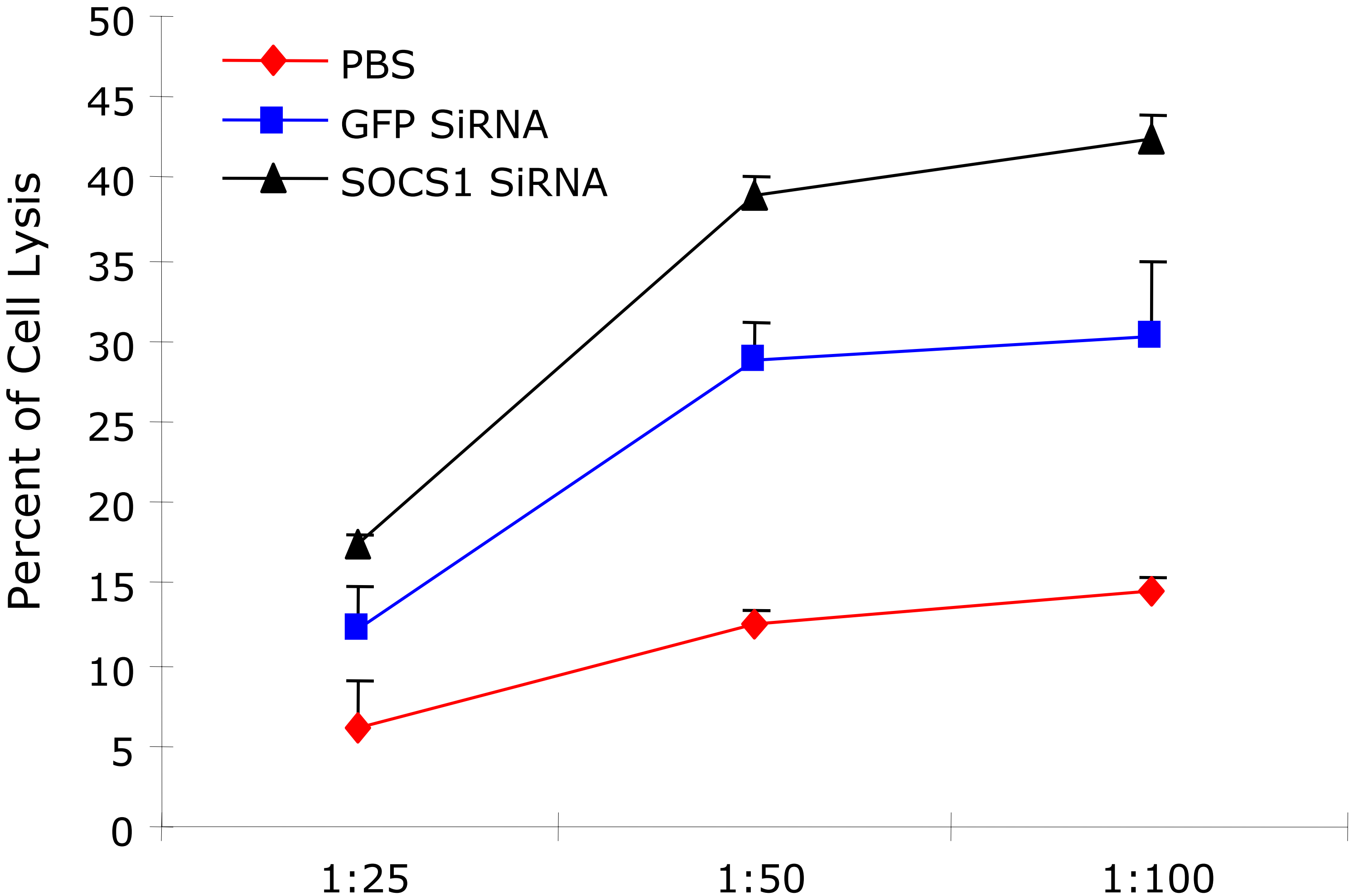

PBS
GFP SiRNA
SOCS1 SiRNA
Percent of Cell Lysis
0
5
10
15
20
25
30
35
40
45
50
1:25
1:50
1:100

S-Fig. 3
