# Supplementary material for: An Alternative and Effective HIV Vaccination Approach Based on Inhibition of Antigen Presentation Attenuators in Dendritic Cells
Source: PLoS Med. 2006 Jan 3;3(1):e11. doi: 10.1371/journal.pmed.0030011 (PMC1323501; doi:10.1371/journal.pmed.0030011)
Supplement: Supplementary file 4 [file pmed.0030011.sg004.pdf]

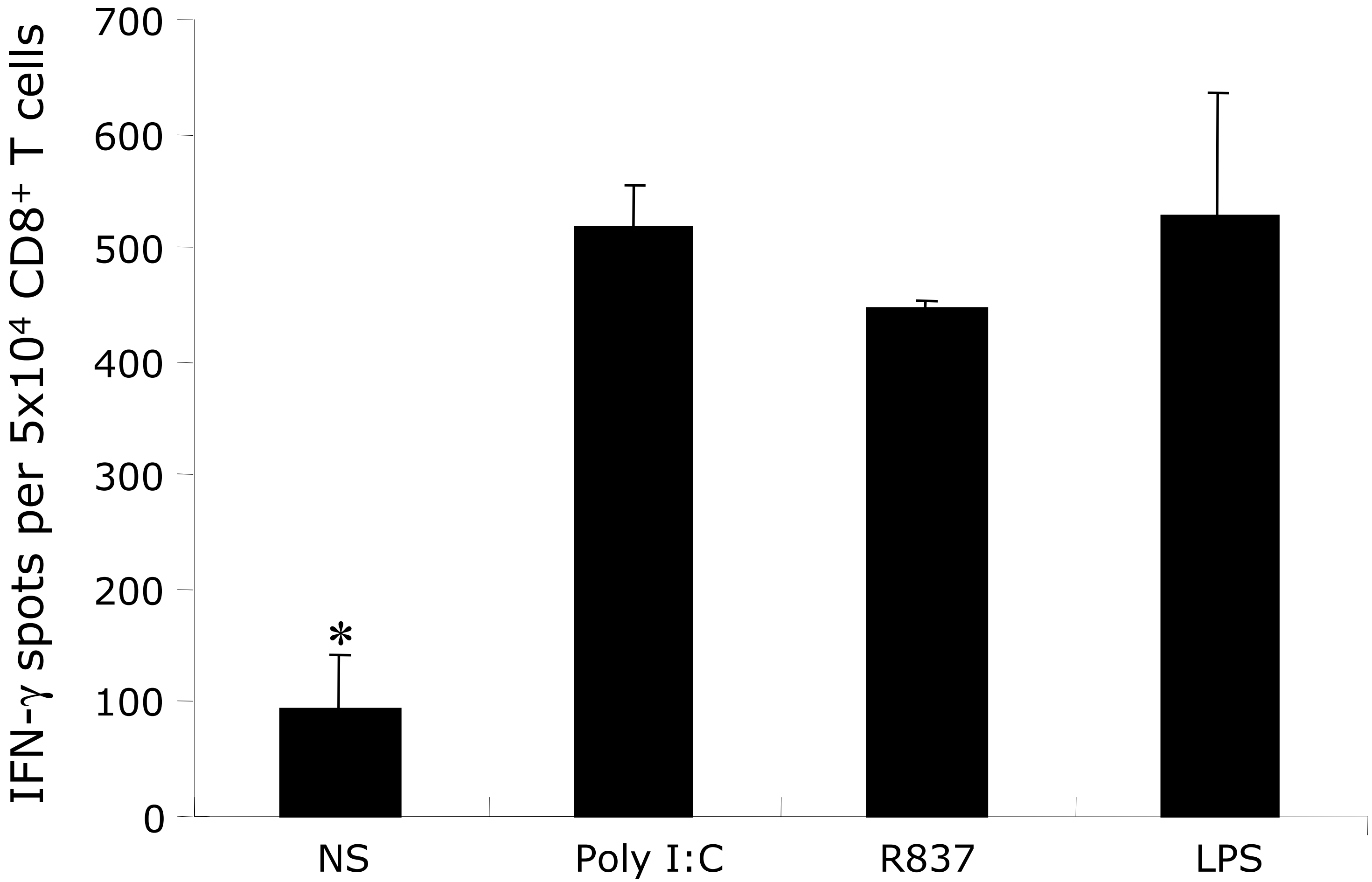

IFN-γ spots per 5x10⁴ CD8⁺ T cells
700
600
500
400
300
200
100
0
*
NS
Poly I:C
R837
LPS

S-Fig. 4
