# Supplementary material for: An Alternative and Effective HIV Vaccination Approach Based on Inhibition of Antigen Presentation Attenuators in Dendritic Cells
Source: PLoS Med. 2006 Jan 3;3(1):e11. doi: 10.1371/journal.pmed.0030011 (PMC1323501; doi:10.1371/journal.pmed.0030011)
Supplement: Supplementary file 5 [file pmed.0030011.sg005.pdf]

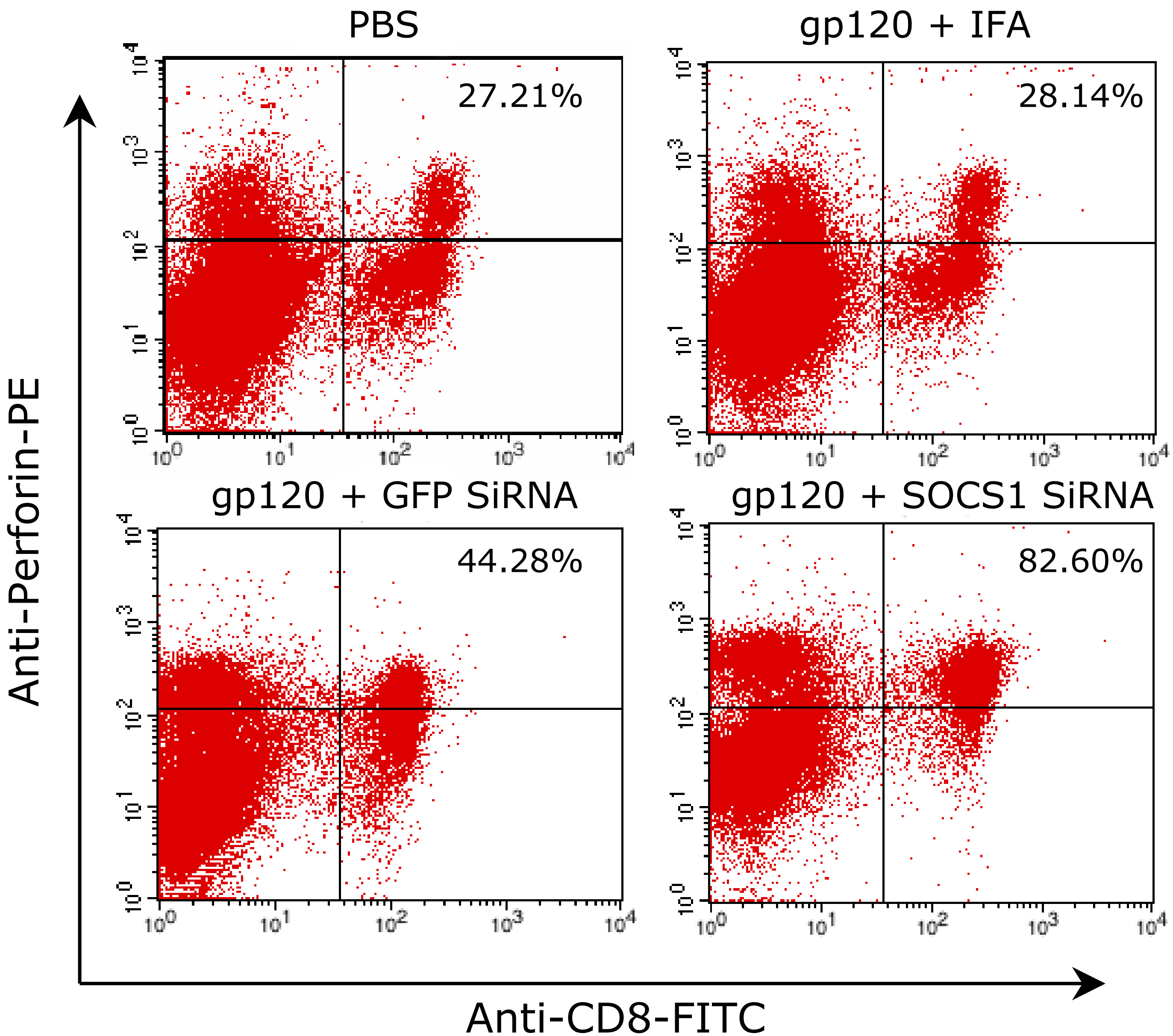
PBS
27.21%
gp120 + IFA
28.14%
gp120 + GFP SiRNA
44.28%
gp120 + SOCS1 SiRNA
82.60%
Anti-Perforin-PE
Anti-CD8-FITC

S-Fig. 6
