# Supplementary material for: An Alternative and Effective HIV Vaccination Approach Based on Inhibition of Antigen Presentation Attenuators in Dendritic Cells
Source: PLoS Med. 2006 Jan 3;3(1):e11. doi: 10.1371/journal.pmed.0030011 (PMC1323501; doi:10.1371/journal.pmed.0030011)
Supplement: Supplementary file 6 [file pmed.0030011.sg006.pdf]

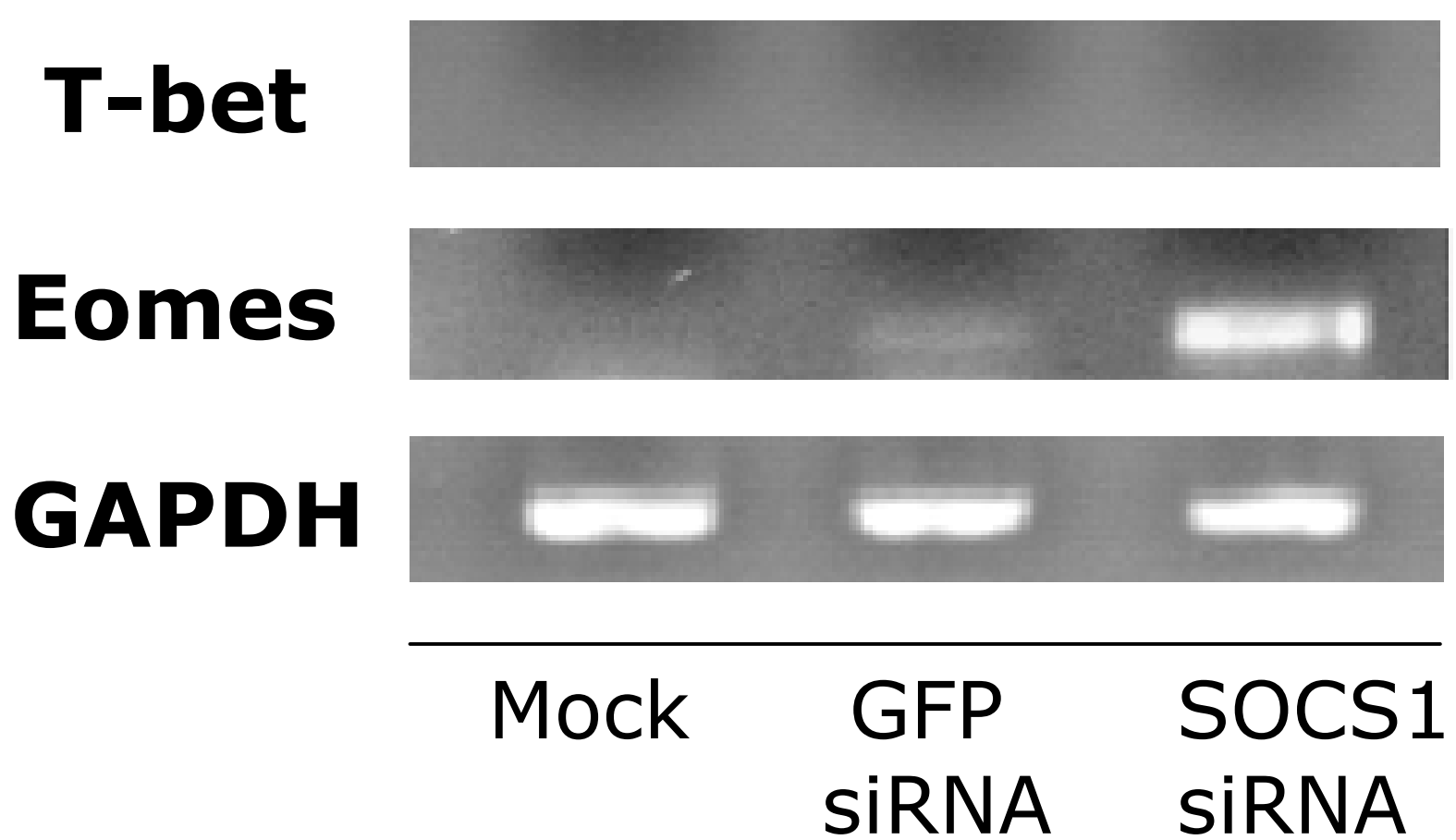

S-Fig. 5
